# DNA Methylation Restricts Lineage-specific Functions of Transcription Factor Gata4 during Embryonic Stem Cell Differentiation

**DOI:** 10.1371/journal.pgen.1003574

**Published:** 2013-06-27

**Authors:** Masaaki Oda, Yuichi Kumaki, Masaki Shigeta, Lars Martin Jakt, Chisa Matsuoka, Akiko Yamagiwa, Hitoshi Niwa, Masaki Okano

**Affiliations:** 1 Laboratory for Mammalian Epigenetic Studies, Center for Developmental Biology, RIKEN, Kobe, Japan; 2 Laboratory for Pluripotent Cell Studies, Center for Developmental Biology, RIKEN, Kobe, Japan; 3 Laboratory for Stem Cell Biology, Center for Developmental Biology, RIKEN, Kobe, Japan; Albert Einstein College of Medicine, United States of America

## Abstract

DNA methylation changes dynamically during development and is essential for embryogenesis in mammals. However, how DNA methylation affects developmental gene expression and cell differentiation remains elusive. During embryogenesis, many key transcription factors are used repeatedly, triggering different outcomes depending on the cell type and developmental stage. Here, we report that DNA methylation modulates transcription-factor output in the context of cell differentiation. Using a drug-inducible Gata4 system and a mouse embryonic stem (ES) cell model of mesoderm differentiation, we examined the cellular response to Gata4 in ES and mesoderm cells. The activation of Gata4 in ES cells is known to drive their differentiation to endoderm. We show that the differentiation of wild-type ES cells into mesoderm blocks their Gata4-induced endoderm differentiation, while mesoderm cells derived from ES cells that are deficient in the DNA methyltransferases Dnmt3a and Dnmt3b can retain their response to Gata4, allowing lineage conversion from mesoderm cells to endoderm. Transcriptome analysis of the cells' response to Gata4 over time revealed groups of endoderm and mesoderm developmental genes whose expression was induced by Gata4 only when DNA methylation was lost, suggesting that DNA methylation restricts the ability of these genes to respond to Gata4, rather than controlling their transcription *per se*. Gata4-binding-site profiles and DNA methylation analyses suggested that DNA methylation modulates the Gata4 response through diverse mechanisms. Our data indicate that epigenetic regulation by DNA methylation functions as a heritable safeguard to prevent transcription factors from activating inappropriate downstream genes, thereby contributing to the restriction of the differentiation potential of somatic cells.

## Author Summary

Animal bodies are constructed from many different specialized cell types that are generated during embryogenesis from a single fertilized egg, and acquire their specific characteristics through a series of differentiation steps. After being committed to a specific cell type, it is generally difficult for differentiated cells to convert to other cell types, at least partly because the cells maintain some memory or mark of their developmental history. Such cellular memory is mediated by “epigenetic” mechanisms, which function to stabilize the cell state. DNA methylation, a chemical modification of genomic cytosine residues, is one such mechanism. Genomic DNA methylation patterns in early embryonic cells are established in a cell-type-dependent manner, and these specific patterns are propagated through cell divisions in a clonal manner. However, our understanding of how DNA methylation controls cell differentiation and developmental gene regulation is limited. In this study, using an *in vitro* model of differentiation, we obtained evidence that DNA methylation modulates the cell's response to DNA-binding transcription factors in a cell-type-dependent manner. These findings extend our understanding of how cellular traits are stabilized within specific lineages during development, and may contribute to advances in cellular engineering.

## Introduction

Development is based on a series of cell-fate decisions and commitments. Transcription factors and epigenetic mechanisms coordinately regulate these processes [1], [2]. Transcription factors play dominant roles in instructing lineage determination and cell reprogramming [3], [4]. Transcription factor and co-factor networks regulate cell-specific gene programs, allowing a given transcription factor to be used repeatedly in different cellular and developmental contexts [5]. In addition, epigenetic mechanisms, which establish and maintain cell-specific chromatin states (or epigenomes) during differentiation and development [6], modulate the functions of transcription factors in cell-type-dependent manners [7], [8]. Alterations of chromatin states can increase the efficiency of transcription factor-induced cell reprogramming [9], [10] and lineage conversion *in vivo* [11], [12]. However, how epigenetic mechanisms and transcription factor networks coordinately regulate cell differentiation remains elusive.

DNA methylation at cytosine-guanine (CpG) sites is a heritable genome-marking mechanism for epigenetic regulation, modulating gene expression through chromatin regulation [13]. Genome-wide DNA methylation profiles have revealed that the methylated CpG in the mammalian genome is specifically distributed in a cell-type-dependent manner [14]–[16], and the methylated CpG sites are dynamically reprogrammed during embryogenesis and gametogenesis [17]–[19]. The DNA methylation profile is established and maintained by three DNA methyltransferases (DNMTs), Dnmt1, Dnmt3a, and Dnmt3b [20], together with DNA demethylation mechanisms [21]. Dnmt1 is required for the maintenance of DNA methylation profiles, whereas Dnmt3a and Dnmt3b are required to establish them. The inactivation of Dnmt1 or both Dnmt3a and Dnmt3b in mice leads to early embryonic lethality, showing that DNA methylation has essential roles in mammalian embryogenesis [22]–[24]. DNA methylation is involved in various cell-differentiation processes, and several studies have identified the underlying mechanisms for specific cases [25]–[29]. However, the roles of DNA methylation in differentiation and development remain largely unexplored.

The evolutionarily conserved zinc-finger transcription factor GATA family controls tissue-specific gene expression and cell-fate determination in many cell types [30]. Gata4 is broadly expressed in endoderm- and mesoderm-derived tissues as well as in pre-implantation embryos. Gata4 functions in endoderm formation, cardiac morphogenesis, the establishment of regional identities in the small intestine, and tissue-specific gene expression in the liver and osteoblasts [31]–[36]. Even though Gata4 has a broad expression profile, it still has cell-specific functions, which are determined largely by transcription factor and co-factor networks. Unique interactions with cardiogenic transcription factors and co-factors allow Gata4 to regulate cardiac gene expression specifically in cardiac progenitor cells and their derivatives [37]. In contrast, the overexpression of Gata4 alone causes mouse ES cells to differentiate into extra-embryonic primitive endoderm cells [38], indicating that Gata4 functions as a master regulatory transcription factor for endoderm specification in ES cells. It is likely that Gata4 is unable to activate a cardiac gene program in ES cells, because of the lack of cardiac transcription factors and co-factors. However, it remains unclear how the endoderm-instructive function of Gata4 is suppressed in non-endoderm tissues, such as mesoderm. Epigenetic mechanisms such as DNA methylation may modulate the cell-specific functions of Gata4.

Here, we have established an *in vitro* experimental system to test the downstream output of Gata4 in two defined cell types, ES and mesoderm progenitor cells, using a drug-inducible Gata4 and an ES-cell differentiation protocol. Using this experimental system, we examined the effect of DNA methylation on Gata4-induced endoderm differentiation and developmental gene regulation during mesoderm-lineage commitment. Our findings suggest that DNA methylation restricts the endoderm-differentiation potential in mesoderm cells and controls the responsiveness of developmental genes to Gata4.

## Results

### Suppression of the Endoderm-Instructive Function of Gata4 in ES-Cells after Differentiation

To explore the role of DNA methylation in the context-dependent function of transcription factors, we focused on Gata4 as a model. Gata4 instructs the primitive endoderm fate in ES cells [38], while it regulates various endoderm and mesoderm tissue-specific genes in somatic cells [30]. In this study, we took advantage of a drug-inducible Gata4 construct where the Gata4 coding region is fused with the ligand-binding domain of the human glucocorticoid receptor (Gata4GR) [39]. The activation of Gata4GR by adding dexamethasone (Dex), a glucocorticoid receptor ligand, drove the differentiation of wild-type (WT) ES cells into the primitive endoderm lineage, in which all the cells were positive for the primitive endoderm marker Dab2 (Figure S1A–S1D, LIF(+) condition). However, when the ES cells were first differentiated for 3 days by withdrawing leukemia inhibitory factor (LIF) from the ES maintenance medium, the cells became resistant to the Gata4-induced endoderm differentiation (Figure S1A–S1D, LIF(−) condition), showing that the endoderm-instructive function of Gata4 is suppressed after somatic cell differentiation.

**Figure 1:**
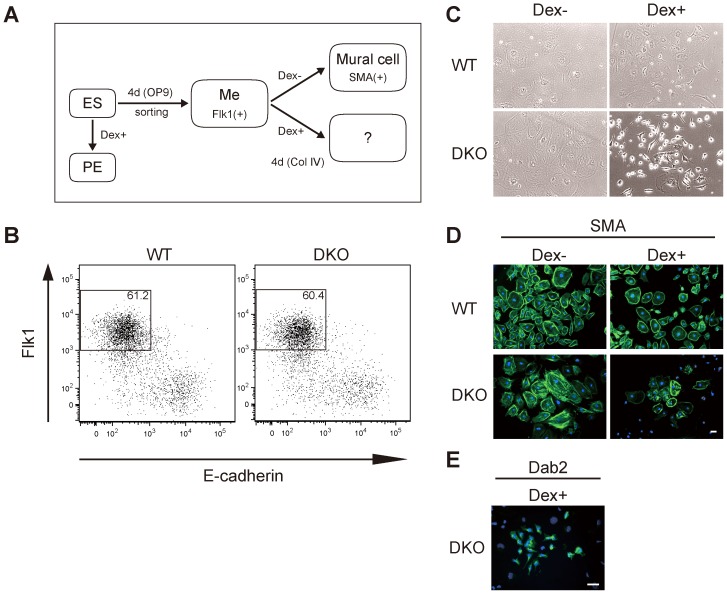
Gata4-induced primitive endoderm differentiation from *Dnmt3a* ^−/−^ *Dnmt3b* ^−/−^ (DKO) Flk1(+) mesoderm cells derived from OP9 co-culture conditions. (*A*) Experimental strategy for isolating mesoderm progenitors from ES cells and the subsequent activation of Gata4. Wild-type (WT) or DKO ES cells stably expressing Gata4GR were differentiated on OP9 stromal cells for 4 days. The Flk1(+) mesoderm cells (Me) were sorted and cultured on type IV collagen with or without dexamethasone (Dex) to activate Gata4GR. ES cells were also directly differentiated into primitive endoderm (PE) by adding Dex to ES maintenance medium containing LIF. (*B*) Flow cytometry profiles of Flk1 and E-cadherin in ES cells differentiated on OP9 stromal cells. The percentages of Flk1(+)/E-cadherin(−) cells are indicated. (*C*) Phase-contrast photomicrographs of differentiated Flk1(+) mesoderm cells. WT or DKO Flk1(+) cells were cultured with or without Dex for 4 days. (*D*, *E*) Immunofluorescence analysis of the mural cell marker SMA (*D*) or endoderm marker Dab2 (*E*) (green) in WT or DKO Flk1(+) mesoderm cells cultured for 4 days with or without Dex. DNA was stained with Hoechst 33342 (blue). All experiments were performed three times. Scale bar, 50 µm.

To investigate the Gata4 response in a defined somatic cell population, we employed a mesoderm differentiation protocol, in which ES cells were co-cultured with OP9 stroma cells [40] without LIF for 4 days and then sorted to isolate the Flk1 (also known as VEGFR2 or KDR)-positive (+) population [41] (Figure 1A). Flk1(+) cells derived from ES cells are considered to be equivalent to a mixture of primitive and lateral mesoderm [41], and these cells can differentiate into several mesoderm derived lineages. To eliminate less differentiated cells (including mesendoderm) and ensure their mesoderm commitment, we isolated the Flk1(+)/E-cadherin(−) population by flow cytometry [42] (Figure 1B).

Flk1(+) mesoderm cells derived from WT ES cells can efficiently differentiate into vascular mural cells, which express alpha-smooth muscle actin (SMA), when cultured on type IV collagen [43] (Figure 1A, 1C, 1D, WT Dex−). When we activated Gata4GR by adding Dex, the WT Flk1(+) mesoderm cells also differentiated into mural cells, and the entire cell population was positive for SMA staining at an intensity similar to that of cells without Gata4 induction, although some of the Gata4-induced cells were noticeably smaller in size (Figure 1C, 1D, WT Dex+). We found no round, Dab2-positive cells among the WT Flk1(+) cells that differentiated with Gata4 induction (data not shown). These results indicated that the endoderm-instructive function of Gata4 was suppressed after the differentiation of ES cells into Flk1(+) mesoderm.

### Primitive Endoderm Differentiation from DNA-Hypomethylated Flk1(+) Mesoderm Cells in Response to Gata4

We next examined whether DNA methylation was involved in the suppression of the endoderm-instructive function of Gata4 in Flk1(+) mesoderm cells. For this, we used *Dnmt3a* ^−/−^ *Dnmt3b* ^−/−^ double-knockout (DKO) mouse ES cells, which have no *de novo* methylation activity and low DNA methylation levels at many loci [24], [44]. DKO ES cells expressing Gata4GR differentiated efficiently from ES cells into primitive endoderm in the presence of Dex, similar to WT ES cells (Figure S2). We obtained the DKO Flk1(+) mesoderm population at the same high efficiency as the WT Flk1(+) cells (Figure 1B), and the DKO Flk1(+) cells differentiated into SMA(+) mural cells with a similar efficiency to WT Flk1(+) cells (Figure 1C, 1D, DKO Dex−), indicating that DNA hypomethylation does not by itself inhibit ES-cell differentiation into Flk1(+) mesoderm and SMA (+) mural cells. We then tested the response of the DKO Flk1(+) mesodermal cells to Gata4 activation (Figure 1A). After 4 days of induction with Gata4, most of the differentiated DKO Flk1(+) cells stained weakly for SMA, although the intensity was somewhat variable (Figure 1D, DKO Dex+). Strikingly, a small number of cells in the population (about 1%) had a round, endoderm-like morphology and were positive for the primitive endoderm marker Dab2 (Figure 1C, 1E, DKO Dex+). Morphologies of the SMA-positive cells (flat and large cytoplasm) and the Dab2-positive cells (round and small cytoplasm) were distinct (Figure 1C–1E). These results indicated that some DKO Flk1(+) mesoderm cells were converted to endodermal identity in response to Gata4.

Dnmt3a and Dnmt3b have transcriptional repression activities that are independent of their enzymatic activities [45], [46]. To examine whether this lineage conversion was DNA methylation-dependent, we prepared Flk1(+) mesoderm cells from *Dnmt1* ^−/−^ (KO) ES cells [23] expressing Gata4GR (Figure S3A), in which DNA methylation in the genome is extensively decreased due to the loss of maintenance methylation activity. Although overall tendencies for low growth and survival were observed, the *Dnmt1* ^−/−^ Flk1(+) cells efficiently differentiated into SMA(+) cells without Gata4 induction, whereas Dab2(+) primitive endoderm-like cells emerged when Gata4 was induced (Figure S3B, S3C). These results indicated that it was the loss of DNA methylation that promoted the Flk1(+) mesoderm cells to convert their lineage to endoderm in response to Gata4. These results also exclude the contribution of clonal effects caused by genetic or epigenetic changes associated with individual cell lines unrelated to DNMT functions.

We observed similar results using DKO Flk1(+) mesoderm cells obtained using a different mesoderm differentiation condition, in which ES cells were cultured on type IV collagen-coated dishes [41] (Figure S4A). Although the recovery of the Flk1(+) population from DKO ES cells was low (3%) under this condition (Figure S4B), the DKO Flk1(+) cells differentiated into SMA-positive mural cells with an efficiency similar to that of the WT Flk1(+) cells (Figure S4C,D, Dex−), confirming the commitment of the DKO Flk1(+) cells to the mesoderm fate. Using this condition, we observed that Gata4 reproducibly induced highly efficient differentiation of DKO Flk1(+) mesoderm cells into the endoderm, in which most cells were Dab2-positive with an endoderm-like, round cell morphology, while no such cells were observed in the WT Flk1(+) cell cultures (Figure S4C, S4D, WT DKO Dex+). We used RT-PCR and microarray analysis to obtain gene expression profiles of these cell populations, and found that when DKO Flk1(+) mesoderm cells were cultured for 4 days with Gata4 activation (DKO Flk1+Dex+), their gene expression profile was similar to that of primitive endoderm cells that were derived directly from ES cells (Figure S4E–S4G).

However, we found that this culture condition was not stable; although it initially gave more efficient Gata4-induced differentiation of DKO Flk1(+) mesoderm cells into the endoderm (Figure S4C, S4D, DKO Dex+) compared to the OP9 co-culture condition (Figure 1C–1E, DKO Dex+), later, the efficiency of the both conditions became similar. We speculated that stroma-cell-free culture systems, such as the type IV collagen condition, may be more sensitive to factors such as the serum lot used in the culture medium. Because of the consistent differentiation properties and the high recoveries of Flk1(+) mesoderm cells, we used the OP9 co-culture condition for mesoderm differentiation in the remaining analyses.

### Transcriptome Analysis of Gata4-Induced, DNA-Hypomethylated Mesoderm Cells

**Figure 2:**
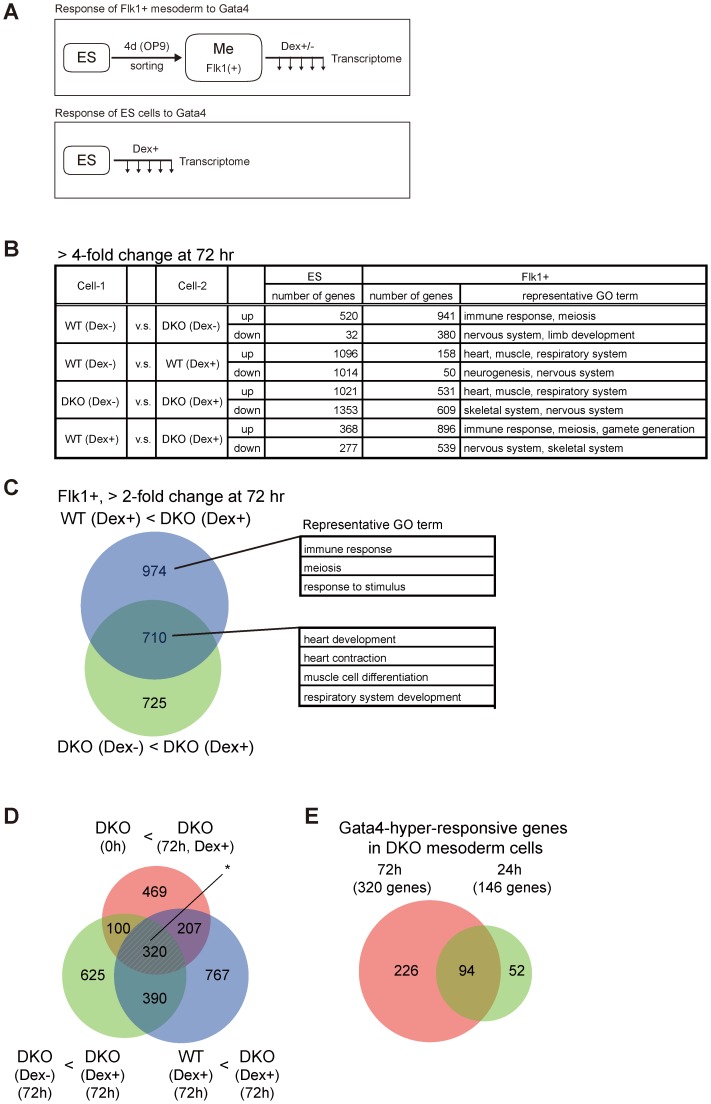
Transcriptome analysis of Gata4-induced DKO Flk1(+) mesoderm cells. (*A*) Experimental scheme to examine transcriptome changes in response to Gata4 in mesoderm or ES cells. WT or DKO ES cells stably expressing Gata4GR were differentiated on OP9 stromal cells for 4 days, then the Flk1(+) mesoderm cells (Me) were sorted and cultured with or without Dex to activate Gata4GR (top). The same WT and DKO ES cells were also cultured with Dex in ES culture conditions (bottom). The expression microarray data were obtained at several time points (up to 72 hr) after Gata4GR activation in both mesoderm and ES cells. (*B*) Numbers of genes with a more than 4-fold difference between the indicated cell conditions 72 hr after Gata4GR activation by the addition of Dex in ES or Flk1(+) mesoderm cells. In each comparison, ‘*up*’ represents genes expressed higher in Cell-2 than Cell-1, and ‘*down*’ represents genes expressed lower in Cell-2 than Cell-1. Representative gene ontology (GO) terms at Biological Process level 4 (BP4) for differentially expressed genes in Flk1(+) mesoderm cells are shown in the right column. (*C*) Venn diagram representing the overlap between (i) the genes expressed 2-fold higher in DKO Flk1(+) mesoderm with Dex at 72 hr than WT cells under the same conditions (WT Dex+<DKO Dex+, purple) and (ii) the genes expressed 2-fold higher in DKO Flk1(+) cells with Dex than the same cells without Dex at 72 hr (DKO Dex−<DKO Dex+, light green). Representative GO terms for genes within the indicated areas are shown. Detailed results of the GO analysis are provided in Tables S1 and S2. (*D*) Extraction of Gata4-hyper-responsive genes in Dnmt3a/Dnmt3b-deficient Flk1(+) mesoderm cells from transcriptome data. Venn diagrams of the 2-fold upregulated genes in DKO mesoderm with Gata4 activation at 72 hr compared to (i) WT cells under the same conditions (WT Dex+<DKO Dex+, purple), (ii) the same cells without Gata4 activation (DKO Dex−<DKO Dex+, light green), or (iii) the same cells immediately after Flk1(+) sorting (DKO 0 h<DKO Dex+, orange). The overlapping genes of these three categories (320 genes, marked with an asterisk) are considered to be the genes that are upregulated in response to Gata4 preferentially at low DNA methylation levels. (*E*) Venn diagram of the Gata4-responsive genes in DNA-hypomethylated mesoderm cells at 72 hr and 24 hr identified in (*D*) and Figure S5. The 94 overlapping genes were used for the analysis in Figure 3, while the 320 genes at 72 hr were used in Figure S6.

The above results indicated that DNA methylation is involved in the suppression of the endoderm-instructive function of Gata4 in mesoderm cells. Thus, we wondered how the DNA-hypomethylated mesoderm cells reprogrammed their transcription profiles from mesoderm to endoderm in response to Gata4. Previous studies in other cell-differentiation models suggested two possibilities: (1) the loss of DNA methylation together with Gata4 activity de-represses a few gatekeeper genes for endoderm differentiation, and these gatekeepers then activate the endoderm transcriptional program [27], [29]; (2) alternatively, the loss of DNA methylation allows Gata4 to directly activate its endoderm downstream genes [26]. To address these possibilities, we dissected the temporal changes of the transcriptome in response to Gata4 in Flk1(+) mesoderm cells (Figure 2A). RNA was isolated at several time points (up to 72 hr) from WT or DKO Flk1(+) cells cultured with or without Gata4 activation, and their genome-wide transcriptional profiles were analyzed using microarrays (Figure 2A, Flk1+ mesoderm). For comparison, we also obtained the temporal transcriptome of ES cells in response to Gata4 at similar time points (Figure 2A, ES).

We first examined how many genes were differentially expressed as a result of the loss of Dnmt3a/Dnmt3b or Gata4 activation at 72 hr (Figure 2B). In total, 941 genes were expressed at a more than fourfold higher level in DKO Flk1(+) mesoderm cells cultured without Gata4 activation, compared to WT cells (Figure 2B, WT Dex− vs. DKO Dex−, up), which may represent genes directly repressed by DNA methylation. The gene ontology (GO) terms related to immune response, meiosis, and gametogenesis were significantly enriched in this gene set (Table S1), which is consistent with a previous report showing that promoters of germline- or inflammation-associated genes are methylated *de novo* during mouse embryogenesis in a Dnmt3-dependent manner [47]. Because DKO Flk1(+) cells without Gata4 activation properly differentiated into mural cells with a cell morphology indistinguishable from that of WT Flk1(+) cells (Figure 1C, 1D), the upregulation of these 941 genes seemed to have little impact on the cellular phenotype of mesodermal differentiation. In contrast, Gata4 activation in both the WT and DKO Flk1(+) cells resulted in differential expression of hundreds of genes, for which the GO terms related to various developmental processes were enriched (Figure 2B, Table S1).

We then extracted the genes that responded to Gata4 preferentially in DKO cells with hypomethylated DNA (Figure 2C–2E, Figure S5). The overlap between (i) the genes expressed more highly in DKO cells than in WT cells with Gata4 induction (WT Dex+<DKO Dex+, >2-fold change) and (ii) the genes expressed more highly in DKO cells with Gata4 induction than in the same cells without Gata4 induction (DKO Dex−<DKO Dex+, >2-fold change) separated the Gata4-responsive genes (710 genes) from the DNA methylation-sensitive genes (974 genes) (Figure 2C, Table S2). Based on the further overlap with (iii) the genes expressed at higher levels in DKO cells after 72 h of mesodermal differentiation with Gata4 induction than in the cells immediately after mesodermal differentiation (DKO 0 hr<DKO Dex+, >2-fold change), we identified 320 genes that responded to Gata4 at the 72 hr time point preferentially on the DKO background in Flk1(+) mesoderm cells (Figure 2D). To extract early response genes to Gata4, we identified the same overlaps in gene sets differentially expressed at 24 hr (146 genes, Figure S5). Based on the overlap of these 146 genes with the Gata4 responsive genes at 72 hr, we identified 94 genes that responded to Gata4 within 24 hr and lasted for at least 72 hr, preferentially on the DKO background, in Flk1(+) mesoderm cells (Figure 2E, Table S3). These results indicated that a significant number of genes became hyper-responsive to Gata4 in DNA-hypomethylated DKO mesoderm cells.

### Time-Course Analysis of Transcriptome Changes in Response to Gata4

**Figure 3:**
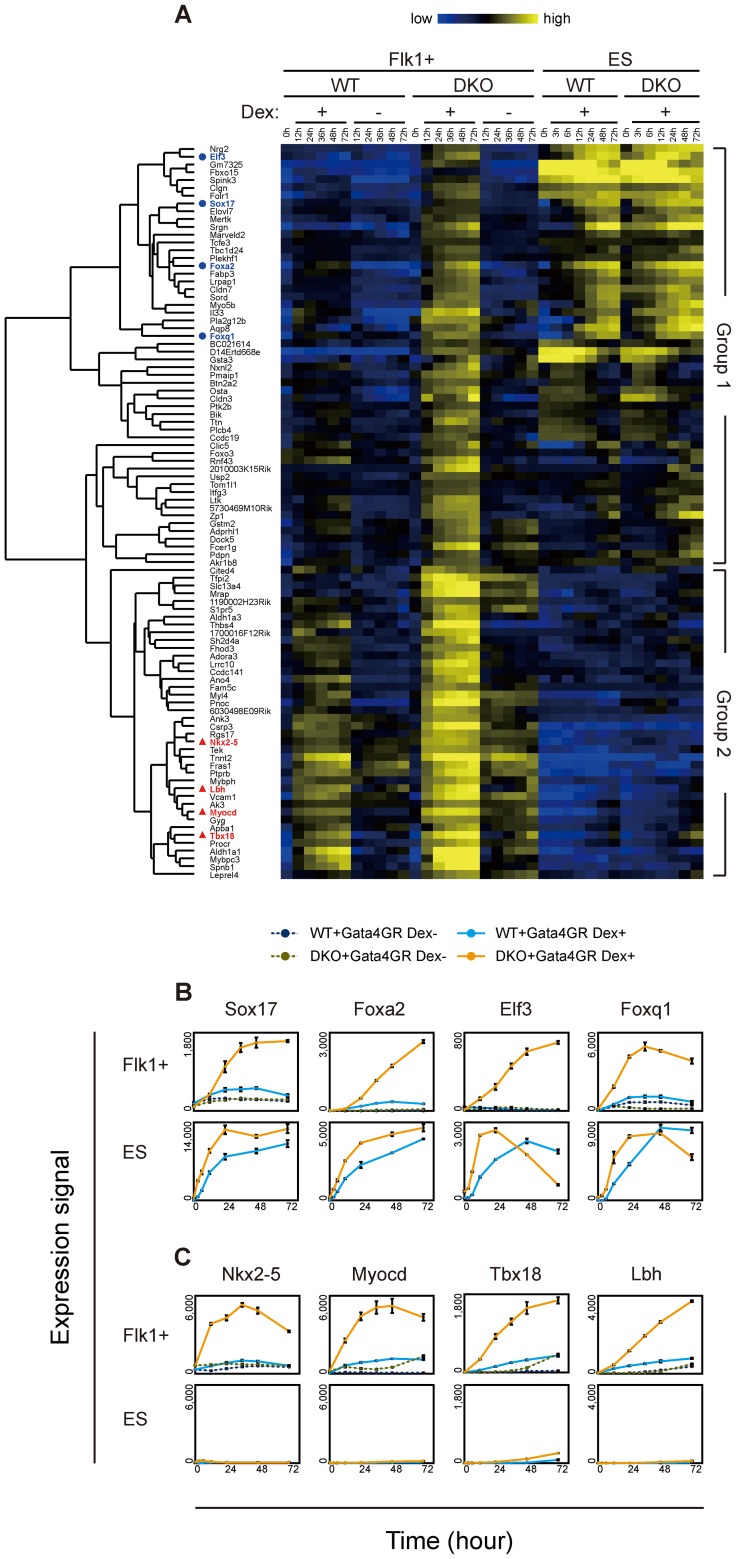
Time course analysis of transcriptome changes in response to Gata4. (*A*) Cluster heat map representing the temporal transcriptional changes for Gata4 response genes in Flk1(+) mesoderm or ES cells. WT or DKO Flk1(+) mesoderm cells or ES cells expressing Gata4GR were cultured for 72 hr in the presence or absence of Dex, and expression microarray data were obtained at several time points (0, 12, 24, 36, 48, and 72 hr for Flk1(+) mesoderm cells; 0, 3, 6, 12, 24, 48, and 72 hr for ES cells). Ninety-four genes whose responses to Gata4 were higher in DKO than WT Flk1(+) mesoderm cells at 24 hr were extracted as described in Figure 2E. The clustering of these 94 genes was based on their temporal expression profiles in Flk1(+) mesoderm and ES cells, and the resulting dendrogram is shown at left. Genes used in (*B*) and (*C*) are highlighted as blue circles and red triangles, respectively. Relative gene expression values (log2) are represented as colors, from lowest (blue) to highest (yellow). (*B*, *C*) Line graphs of the gene expression values (linear) from the microarray data. The mean values of triplicates (Flk1+, 0 hr and Dex+) or duplicates (others) with their standard deviations are shown. (*B*) Ectopic expression of endoderm genes in response to Gata4 in DKO mesoderm cells. These genes responded to Gata4 in WT and DKO ES cells, but not in WT mesoderm cells. Note that smaller scales are used for the expression signal for Flk1(+) mesoderm cells compared to those for ES cells. (*C*) Precocious expression of cardiac genes in response to Gata4 in DKO mesoderm cells. These genes did not respond to Gata4 in WT or DKO ES cells. “WT+Gata4GR Dex+” and “WT+Gata4GR Dex−”, WT cells expressing Gata4GR with and without Dex, respectively; “DKO+Gata4GR Dex+” and “DKO+Gata4GR Dex−”, DKO cells expressing Gata4GR with and without Dex, respectively.

We then examined the time course of the expression profiles of these Gata4-hyper-responsive genes in Flk1(+) mesoderm and ES cells with or without Gata4 induction (Figure 3A, Figure S6). These Gata4-hyper-responsive genes were divided into two groups by hierarchical clustering (Table S3): group 1 genes responded to Gata4 in ES cells (upper part, Figure 3A, Figure S6), whereas group 2 genes did not (lower part, Figure 3A, Figure S6).

Consistent with the endoderm-instructive function of Gata4 in ES cells [38], group 1 contained many genes expressed in endoderm-derived tissues, such as the liver, intestine, and stomach (BioGPS, http://biogps.org/) [48]. Endoderm transcription factor genes (*Sox17*, *Foxa2*, *Elf3*, *Foxq1*) as well as endoderm lineage-specific genes (*Aqp8*, *Sord*, *Akr1b8*, *Pga5*) were upregulated in response to Gata4 in the DKO Flk1(+) mesoderm cells to the same extent as in ES cells (Figure 3B, Figure S7A). In addition, several genes whose expression was not restricted to endoderm-derived tissues (as listed in BioGPS) showed a similar expression profile (Figure S7B). It should be noted that the group 1 genes showed almost no response to Gata4 in WT Flk1(+) mesoderm cells (Flk1+ WT Dex−, Figure 3A, Figure S6). These results suggest that the upregulation of group 1 genes represents the ectopic activation of the endoderm genetic program in the DNA-hypomethylated DKO mesoderm in response to Gata4.

Endoderm transcription factor genes *Sox7* and endogenous *Gata4*, which also have mesoderm functions, were expressed in the Flk1(+) mesoderm cells before Gata4 activation, but their expression remained only in the DKO Flk1(+) mesoderm cells in response to Gata4 (Figure S7D). Interestingly, several primitive endoderm genes (*Hnf4a*, *Fgfr4*, *Amn*, *S100g*) responded to Gata4 specifically in the DKO mesoderm, but the extent of their response was modest compared to that in the ES cells (Figure S7C). No detectable response of other primitive endoderm genes, such as *Gata6* and *Snai1*, to Gata4 was observed in the DKO mesoderm (Figure S7E).

Group 2 contained many genes involved in heart development and function. Cardiac transcription factor (*Nkx2-5*, *Myocd*, *Tbx18*, *Lbh*, *Tbx5*) and heart-specific genes (*Ednra*, *Tnnt2*, *Fhod3*, *Acaa2*, *Ryr2*, *Kcnj5*) were upregulated in response to Gata4 preferentially in the DKO Flk1(+) mesoderm within 24 hr (Figure 3C, Figure S7F). In addition, several genes that are highly expressed in skeletal muscles or osteoblasts (*Fbxo32*, *Thbs4*, *Gyg*, *Pdlim3*, *Leprel4*) showed a similar response in the DKO Flk1(+) mesoderm cells (Figure S7G). These results are consistent with the cardiac and other mesodermal functions of Gata4 [36], [37]. Because Gata4 regulates cardiac genes through its cooperation with other cardiac transcription factors and co-factors [37], it is likely that the cardiac genes did not respond to Gata4 in ES cells because of the lack of such co-factors.

In contrast, since Flk1(+) mesoderm includes cardiac progenitors [49], Flk1(+) cells may be competent to activate the expression of cardiac genes. Consistent with this idea, unlike the group 1 genes, the group 2 genes, including the cardiac genes, responded weakly to Gata4 in WT Flk1(+) mesoderm cells (Flk1+, WT Dex+, Figure 3A, Figure S6). These results suggested that the response of the cardiac genes in group 2 represents the precocious expression of the cardiac gene program in DNA-hypomethylated DKO Flk1(+) mesoderm cells. In addition, group 2 contained genes highly expressed in endoderm-derived tissues (*Tspan8*, *Aldh1a1*, *Psen2*; Figure S7H), which may represent the ectopic activation of the definitive endoderm program.

**Figure 4:**
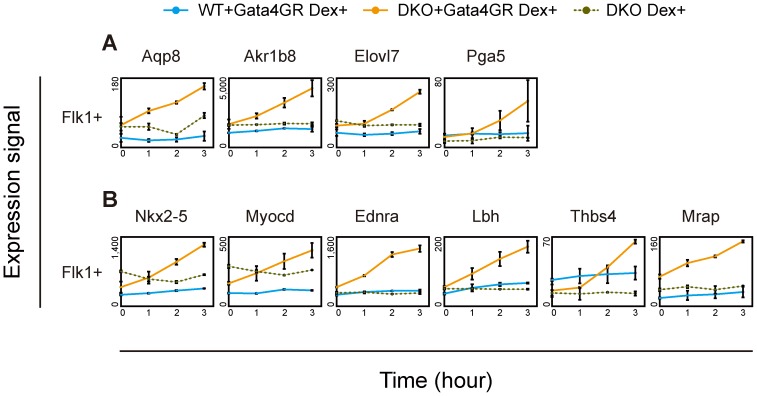
Immediate response to Gata4 in DKO Flk1(+) mesoderm cells. ES cells differentiated on OP9 stromal cells were treated with Dex for 0, 1, 2 or 3 hr. The Flk1(+) mesoderm cells were then sorted by flow cytometry, and their gene expression was analyzed using microarrays. The mean values of duplicates for gene expression values (linear) with their standard deviations are shown. (*A*) Endodermal Gata4-responsive genes. (*B*) Cardiac Gata4-responsive genes. “WT+Gata4GR Dex+”, WT cells expressing Gata4GR with Dex; “DKO+Gata4GR Dex+”, DKO cells expressing Gata4GR with Dex; “DKO Dex+”, DKO cells without the Gata4GR transgene with Dex.

To determine whether the loss of Dnmt3a/Dnmt3b permits Gata4 to directly activate downstream target genes, we examined the immediate response to Gata4 activation by analyzing transcriptome changes occurring within 3 hr. Within the entire transcriptome, the expression of 64 genes were significantly increased, by more than 2-fold, 3 hr after Gata4 activation in DKO Flk1(+) mesoderm cells. Fifteen of these genes overlapped with the Gata4-hyper-responsive genes identified in Figures 2D and S5 (data not shown). Among them, both group 1 genes (*Aqp8*, *Akr1b8*, *Elovl7*, *Pga5*, Figure 4A) and group 2 genes (*Nkx2-5*, *Myocd*, *Ednra*, *Lbh*, *Thbs4*, *Mrap*, Figure 4B) immediately responded to Gata4 in the DNA-hypomethylated DKO mesoderm cells, but not in the WT cells. We also confirmed these results by RT-qPCR (Figure S8). These results suggested that DNA methylation contributes to suppress Gata4 from directly activating these genes.

### Gata4-Binding-Site Profiles and DNA Methylation Analysis in Mesoderm Cells

To gain insight into how DNA methylation modulates Gata4 activation, we examined the Gata4-binding sites in WT and DKO Flk1+ mesoderm cells by ChIP and high-throughput sequencing (ChIP-seq), using anti-Gata4 antibodies. To obtain a large number of cells for the ChIP-seq experiment, WT or DKO ES cells expressing Gata4GR were differentiated in a large-scale culture on OP9 stroma cells, and the Flk1(+) cells were purified by magnetic-activated cell sorting. Gata4 was activated for 3 hr before the cell purification by adding Dex. As controls, WT and DKO ES cells expressing Gata4GR but without Dex treatment were subjected to the analysis. We identified 20,410 peaks for WT Gata4-activatd Flk1(+) cells and 22,733 peaks for DKO Gata4-activated Flk1(+) cells using the DNAnexus software tools.

**Figure 5:**
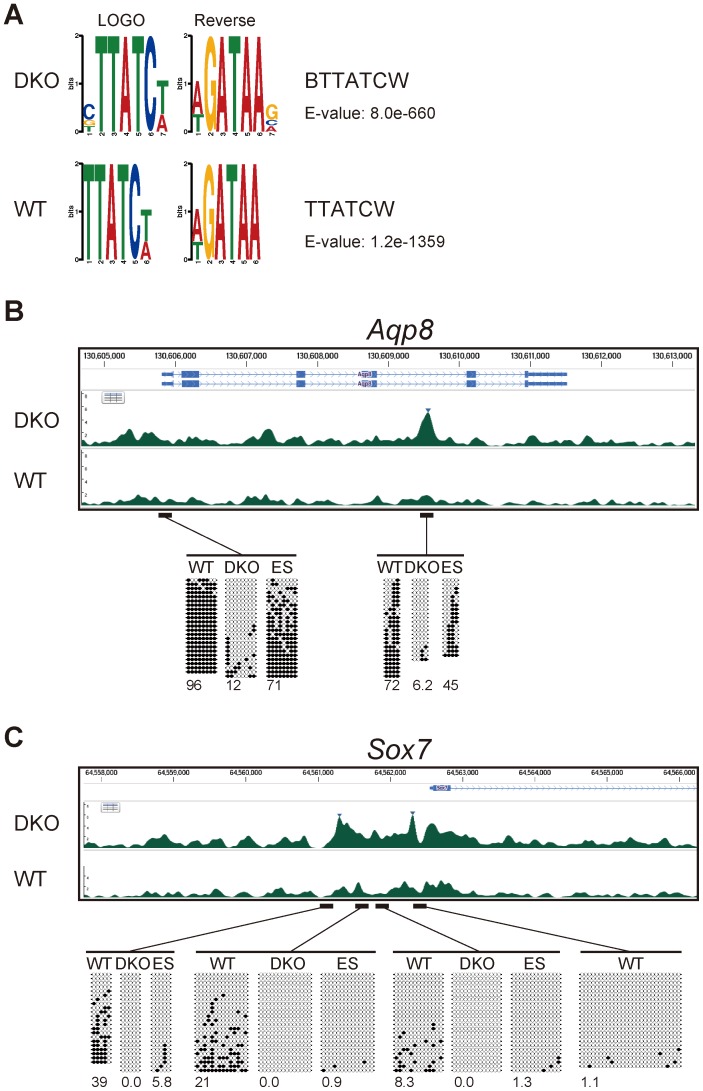
Gata4-binding site and DNA-methylation analyses of Gata4-response genes. (*A*) Motif discovery of transcription-factor-binding motifs by the DREME algorithm using all the peaks of the ChIP-seq data for WT or DKO Flk1(+) cells in which Gata4GR was activated by Dex addition. Logos for the most enriched motif identified by DREME and its reverse complement sequence, motif IDs, and E-values are shown. (*B,C*) Gata4 ChIP-seq enrichment at the Gata4-response gene (*B*) *Aqp8* and (*C*) *Sox7* loci in WT or DKO Flk1(+) cells in which Gata4GR was activated by Dex, and the DNA methylation state at the transcription-start sites and Gata4-binding sites. Tracks represent the mapped read enrichment as determined by DNAnexus software. Blue arrowheads mark Gata4 peaks enriched in DKO Flk1(+) cells compared to WT Flk1(+) cells. Above the peak profiles, the nucleotide positions and Refseq genes are indicated. Horizontal bars represent the genomic regions subjected to DNA methylation analysis by bisulfite sequencing. Open circles represent unmethylated CpGs, and filled circles represent methylated CpGs. The percentage of total CpGs that were methylated is shown below each bisulfite sequencing profile. “WT”, WT Flk1(+) cells; “DKO”, DKO Flk1(+) cells; “ES”, WT ES cells.

To validate the Gata4-ChIP-seq peaks, we performed two independent motif analyses in the MEME Suite software package (http://meme.nbcr.net/) [50]. Using the JASPAR CORE vertebrate motifs (http://jaspar.genereg.net/) [51] and UniPROBE mouse transcription factor motifs (http://thebrain.bwh.harvard.edu/uniprobe/) [52], *ab initio* motif discovery analysis by DREME [53] identified the most highly enriched motifs in the Gata4-ChIP-seq peak regions from both WT and DKO Flk1(+) cells with Gata4 activation as Gata factor-binding motifs (Figure 5A). Similarly, central motif enrichment analysis by CentriMo [54], which assumes that the direct DNA-binding sites tend toward the center of the ChIP-seq peak region, identified the three most highly ‘centrally enriched’ motifs as Gata-factor-binding motifs, using the same motif databases (Figure S9). These results indicated that Gata4-binding sites were highly enriched in the Gata4-ChIP-seq peaks.

We next examined the Gata4 peaks and DNA methylation states of individual Gata4-response genes. Among 146 genes that transcriptionally responded to Gata4 within 24 hours specifically in DKO Flk1(+) mesoderm cells (Figure S5), 70 were associated with the Gata4 peaks in DKO Flk1(+) mesoderm cells, and 52 were associated with DKO-specific Gata4 peaks within a 5-kb distance (data not shown). We then searched for the genes in which either promoter regions [55] or Gata4 peak regions were differentially methylated between WT and DKO mesoderm cells and/or between ES and mesoderm cells, by bisulfite sequencing analysis.

*Aqp8* has a low-CpG promoter, and a DKO-specific Gata4 peak was observed in its intronic region in DKO-mesoderm cells (Figure 5B). Gata4 also bound to the same intronic region in WT ES cells in response to Gata4 activation as revealed by ChIP-qPCR (Figure S10). Both regions were highly methylated in WT but not in DKO mesoderm cells. Thus, the DNA methylation of these regions might affect their Gata4-binding ability or the downstream response of Gata4. However, these regions were moderately or highly methylated in WT ES cells, in which *Aqp8* responded to Gata4 (Figure S7). Thus, the DNA methylation in these regions may have different functions between ES and differentiated somatic cells, as observed in the retrotransposon IAP [56]. *Sox7* has a high-CpG promoter, and DKO-specific Gata4 peaks were observed at this promoter region (Figure 5C). While the 5′-upstream and promoter region of *Sox7* was unmethylated at the undifferentiated ES cell stage, this region was *de novo* methylated, highly at the distal part, during mesoderm differentiation. Similar *de novo* methylation during mesoderm commitment and inverse correlation with Gata4 peaks were observed at the *Cldn7* locus (data not shown). The Gata4-responsive endoderm gene *Lgmn* was associated with two DKO-specific Gata4 peak regions that were highly methylated in Flk1(+) mesoderm cells (Figure S11A). One of the Gata4 peaks, located in the 3′ region of the neighboring gene *Rin3*, was methylated *de novo* during mesoderm differentiation. Since *Rin3* itself did not respond transcriptionally to Gata4, the Gata4 peak located at *Rin3* may contribute to *Lgmn*'s transcription. *Mrap* and *Thbs4*, which have high-CpG promoters, were associated with Gata4 peaks within the gene or the neighboring gene in both WT and DKO mesoderm cells (Figure S11B, S11C). The promoter of *Mrap* was heavily methylated in a Dnmt3-dependent manner, consistent with a previous study [16], while the promoter of *Thbs4* was *de novo* methylated during mesoderm differentiation. These promoter methylations may modulate downstream response of these genes in response to Gata4 binding. We also confirmed by luciferase reporter assay that at least some short fragments associated with Gata4 peaks had enhancer activities in response to Gata4 activation (Figure S12). Note that we also observed DKO-specific Gata4 peak regions that remained locally unmethylated in WT mesoderm cells (data not shown). These Gata4 peaks may represent cooperative binding with other Gata4 molecules or co-factors, or higher-order chromatin state changes induced by a decrease in DNA methylation. Taken together, the Gata4-peak and DNA-methylation profiles suggested that DNA methylation modulates cellular responses to Gata4 through diverse mechanisms.

Collectively, our results show that a significant number of developmental genes, including transcription factors and terminal differentiation genes, were promptly and simultaneously activated in DKO mesoderm cells by Gata4. This finding supports the model in which DNA methylation globally restricts the responsiveness of downstream genes to Gata4, rather than controlling a few gatekeeper genes.

## Discussion

In this study, we characterized the role of DNA methylation in the output of the single transcription factor Gata4 in defined cell types using an ES-cell differentiation method. Mesoderm cells derived from Dnmt3a/Dnmt3b-deficient ES cells were hyper-responsive to Gata4 and activated inappropriate developmental programs. Gata4 induced ectopic expression of endoderm downstream genes and precocious activation of cardiac and other downstream genes in DNA-hypomethylated mesoderm cells; these genes do not respond or respond only weakly to Gata4 in WT cells, suggesting that inappropriate Gata4 target genes such as endoderm genes are repressed in a DNA methylation-dependent manner. Our results indicate that epigenetic regulation by DNA methylation ensures the proper spatial and temporal developmental gene regulation by Gata4 and stabilizes differentiated cellular traits against the possible influences of natural fluctuation or environmental perturbations.

We showed that a fraction of Dnmt3a/Dnmt3b-deficient mesoderm cells, but not WT cells, can convert to endoderm cells in response to Gata4. This effect could be attributable to de-differentiation or the response of a small population of immature cells. However, our data suggest that these possibilities are unlikely. First, we purified the Flk1(+)/E-cadherin(−) cells by flow cytometry, which removes the immature mesendoderm population [42]. Second, Gata4 globally induced endoderm genes in Dnmt3a/Dnmt3b-deficient mesoderm cells, and some genes were expressed at the same level as in Gata4-activated ES cells, the whole population of which differentiates to endoderm. Third, many endoderm genes responded to Gata4 within 12 hr in Dnmt3a/Dnmt3b-deficient mesoderm, and some even responded within 3 hr. These results suggested that Gata4 globally and promptly activates an endoderm gene program in a large population of Flk1(+) mesoderm cells on the Dnmt3a/Dnmt3b-deficient background, but not in WT cells. Thus, it is unlikely that relatively slow processes such as de-differentiation or reversion to pluripotent states [57] are involved in this endoderm differentiation.

Using the mesoderm cells differentiated with OP9 stroma cell co-culture, we found that only a small fraction of the cultured mesoderm cells was differentiated into endoderm based on Dab2 staining (∼1%), even though the expression of endodermal genes were significantly increased (Figure 3, Figure S6, Figure S7). This implies that some cells retaining the mesodermal phenotype express both endoderm and mesoderm genes. During transcription factor induced-somatic cell reprogramming to pluripotent cells, partially reprogrammed cell clones express both stem cell-related genes and lineage-specific genes together [10]. We suggest a model in which an endoderm gene program is activated in a large mesoderm population, priming it for endoderm differentiation, but only a small subset of this population accomplishes the primitive endoderm differentiation.

We showed that the loss of DNA methylation allowed mesoderm cells being converted to endoderm cells in response to Gata4 using both Dnmt1-deficient and Dnmt3a/Dnmt3b-deficient mesoderm cells. However, the low efficiency and incomplete reprogramming of the conversion suggest that additional mechanisms such as cell-specific trans-factors or other epigenetic signatures [58], [59] may also restrict the Gata4-induced endoderm differentiation in Flk1(+) mesoderm cells. These other mechanisms may be coordinately regulated or maintained by DNA methylation. We obtained variable ratios of endoderm differentiation from Gata4-activated Dnmt3a/Dnmt3b-deficient Flk1(+) mesoderm cells when we used the stroma cell-free condition with type IV collagen-coated dishes for mesoderm cell formation (Figure S4 and data not shown). This variation in differentiation efficiency may be due to effects of such restriction mechanisms other than DNA methylation. It is possible that differentiation conditions without stroma cells are more sensitive to various factors in cell culture, which may affect gene expression or epigenetic signatures of the Flk1(+) mesoderm cells differentiated with this condition.

DNA methylation restricts cell differentiation potential during development. Trophectoderm differentiation is restricted in mouse ES cells, and DNA methylation is involved in this process through the DNA methylation-dependent silencing of trophectoderm transcription factor Elf5 [27]. Pancreatic β cell identity is maintained by DNA methylation-dependent silencing of the lineage-determining transcription factor Arx [29]. In these cases, DNA methylation suppresses a limited number of gatekeeper transcription factors. The loss of DNA methylation de-represses these transcription factors, which subsequently activate their downstream transcriptional programs. In contrast, we showed that, in our cellular models, DNA methylation stabilizes mesoderm identity during cell differentiation by restricting the responsiveness of downstream genes to the transcription factor Gata4.

We found that the induction of Gata4 together with the loss of DNMTs, but not Gata4 alone, activates the endoderm gene program in mesoderm cells and promotes endoderm differentiation. During this process, Gata4 promptly activates many endoderm genes, including both transcription factors and terminal differentiation genes with a similar time frame, suggesting that DNA hypomethylation allows Gata4 to activate endodermal target genes directly in mesoderm cells. However, we cannot exclude the possibility that endoderm transcription factors such as Sox17 and Foxa2, which respond to Gata4 early, may contribute more than other proteins to the endoderm differentiation phenotype.

Our results also showed that the loss of DNA methylation alone does not induce endoderm differentiation, showing that the role of DNA methylation is permissive, not instructive, in this differentiation. This is consistent with a previous study of astrocyte differentiation showing that DNA methylation suppresses the responsiveness of embryonic neuroepithelial cells to the gliogenic LIF signal during mouse embryogenesis [26]. The binding of the transcription factor STAT3 to the promoter region of the astrocyte gene *Gfap* is suppressed in a DNA methylation-dependent manner. Similarly, DNA methylation restricts the responsiveness of neuroepithelial cells to Notch signaling by suppressing the binding of the transcription factor RBP-J to the *Hes5* gene promoter [60]. These reports suggest that regulation of the responsiveness of downstream genes to transcription factors is likely to be a broadly used mechanism of DNA methylation-dependent gene regulation. In hematopoietic stem cells (HSCs), a deficiency of Dnmt1 results in impaired self-renewal and skewed myeloid/lymphoid differentiation [61], [62], whereas the inactivation of Dnmt3a leads to an increase in self-renewal associated with the incomplete repression of HSC genes such as *Runx1* [63]. Thus, it is likely that DNA methylation modulates cellular differentiation by multiple pathways and mechanisms.

Several transdifferentiation studies have shown that one or a few transcription factors are sufficient to convert somatic cell fate within several days [3], [64]–[67]. It is likely that there are many genes downstream of transcription factors initiating trans-differentiation, but this aspect has yet to be analyzed in depth. Our study focused on the initial processes during cell fate conversion, and uncovered the contribution of DNA methylation in restricting the global response to the single transcription factor Gata4. Several studies have also suggested a link between DNA methylation and transcription factor-induced cell reprogramming to pluripotency (i.e. iPS cells) [4]. DNA methylation and de-methylation are closely correlated with the epigenetic memory of the original donor cells, and this may contribute to the variable differentiation propensity of iPS cells [68]–[71]. In addition, overall efficiency of the reprogramming process can be improved when somatic cells are treated with DNMT inhibitors [10]. Although, the reprogramming to pluripotency is different from Gata4-induced transdifferentiation in that it requires a much longer period of time, DNA methylation-dependent mechanisms similar to those described here may be involved in the reprogramming process.

DNA methylation regulates gene expression by various mechanisms [72]. The promoter regions of germ-cell-specific genes, inflammation-response genes, and some tissue-specific genes are methylated *de novo* by Dnmt3a/Dnmt3b around the implantation stage of mouse embryogenesis. The expression of these genes is increased by the loss of DNA methylation, indicating that DNA methylation directly represses their transcription [47]. In contrast, in neuronal progenitor cells, the gene body region of neural genes is methylated in a Dnmt3-dependent manner, and the expression of these genes is decreased by the loss of DNA methylation, suggesting that DNA methylation is required to maintain the expression of these genes [73]. Whole-genome DNA methylation analysis showed that the DNA methylation state of distal regulatory enhancers changes dynamically and is linked to changes in the expression of adjacent genes [16], and that the DNA methylation changes of enhancers are driven by transcription-factor binding [16], [74].

In this study, we showed that groups of developmental genes downstream of Gata4 become hyper-responsive to this transcription factor on a Dnmt3a/Dnmt3b-deficient background. The loss of DNA methylation together with Gata4 activation induces the expression of these genes, but the loss of DNA methylation alone does not alter their expression, indicating that DNA methylation does not directly regulate the transcription of these genes. This finding implies that the transcriptome for a given methylome depends on the composition of the transcriptional regulators in a cell. This notion is consistent with previous reports that genome-wide DNA methylation profiles are not well correlated with gene expression [15], [63]. Further mechanistic studies will be necessary to connect the DNA methylome to cellular phenotypes.

In conclusion, our results extend our understanding of the role of DNA methylation in cell differentiation and the stabilization of cellular traits. Together with its feature of clonal inheritance [75], DNA methylation is likely to function as a memory of a cell's developmental history. Elucidation of the mechanisms of DNA methylation targeting and its interaction with chromatin may provide insight into the role of epigenetic regulation in development and cellular reprogramming.

## Materials and Methods

### Cell Lines and Culture

*Dnmt3a^−/−^Dnmt3b^−/−^* DKO ES cells (clone 16aabb), *Dnmt1^−/−^* ES cells (clone 36), and WT J1 ES cells were described previously [23], [24]. WT, DKO, and *Dnmt1^−/−^* ES cell clones stably expressing dexamethasone (Dex)-inducible Gata4 were generated by introducing by electroporation an expression plasmid for Gata4 fused with the ligand-binding domain of the human glucocorticoid receptor (Gata4GR) driven by the CAG promoter [39], followed by selection with L-histidinol dihydrochloride (HisD) (clones J1G4.211, 16G4.3, and 36G4.3, respectively). ES cells were maintained on gelatinized culture dishes in either ES medium, consisting of Glasgow Minimum Essential Medium (GMEM, Sigma) supplemented with 10% fetal calf serum (FCS), 0.1 mM nonessential amino acids (Invitrogen), 1 mM sodium pyruvate, 0.1 mM 2-mercaptoethanol, and 2000 U/ml LIF, or the same medium except for the replacement of 10% FCS with 10% Knockout Serum Replacement (KSR, Invitrogen) and 0.5% FCS. OP9 stromal cells, kindly provided by Dr. Shin-ichi Nishikawa, were maintained in α-Minimum Essential Medium (α-MEM, Invitrogen) supplemented with 20% FCS.

### ES Cell Differentiation

For *in vitro* differentiation by LIF withdrawal, ES cells were cultured overnight in ES medium containing 10% FCS, then differentiation was induced by replacing the medium with medium lacking LIF, after a wash with phosphate-buffered saline (PBS). For primitive endoderm (PE) differentiation, 100 nM Dex was added to ES cells stably expressing Gata4GR, in ES medium containing 10% FCS for 4 days [39]. For the time-course analysis, the cells were recovered by trypsinization at the indicated times after the addition of Dex, and RNA was isolated. For Flk1(+) mesoderm differentiation, ES cells were cultured either on type IV collagen-coated dishes or on OP9 stromal cells [40], [41].

For the type IV collagen-coated dish method, 1×10^5^ WT ES cells or 5×10^5^ DKO ES cells were plated on a type IV collagen-coated 10-cm dish (BioCoat, BD Biosciences) in differentiation medium (α-MEM supplemented with 10% FCS and 50 µM 2-mercaptoethanol) and cultured for 4 days. The cultured cells were then collected using 0.25% trypsin-EDTA, and single-cell suspensions were stained using an allophycocyanin (APC)-conjugated anti-Flk1 antibody (AVAS12, eBioscience), a biotinylated anti-PDGFRα antibody (APA5, eBioscience), and phycoerythrin-conjugated streptavidin (eBioscience). For the OP9 stroma co-culture method, 2×10^5^ WT ES cells or 2.4×10^5^ DKO or *Dnmt1* ^−/−^ ES cells were plated on a 10-cm dish with confluent OP9 stromal cells in the differentiation medium for 4 days.

The cultured cells were collected using 0.25% trypsin-EDTA, and single-cell suspensions were stained using an APC-conjugated anti-Flk1 antibody, a biotinylated anti-E-cadherin antibody (Eccd2, TaKaRa Bio or DECMA-1, eBioscience), and phycoerythrin-conjugated streptavidin. Flk1(+), Flk1(+)/PDGFRα(+), and Flk1(+)/E-cadherin(−) cells were sorted by a FACSAria (BD Biosciences), and the flow cytometry profiles were visualized with FlowJo software (Tree Star). The sorted cells were further cultured on type IV collagen-coated dishes in differentiation medium in the absence or presence of 100 nM Dex for 4 days or the indicated times. For the short-term Gata4-response experiment, ES cells were plated on a 10-cm dish with confluent OP9 stroma cells and cultured for 4 days as described above. One, two, or three hours before cell collection, 100 nM Dex was added to the cell culture. The cells were collected by trypsinization, and the Flk1(+)/E-cadherin(−) cells were sorted as described above. The sorted cells were directly used for RNA isolation.

### Immunofluorescence Analysis

Cells grown on gelatin- or type IV collagen-coated dishes were washed in PBS, fixed with 4% paraformaldehyde for 10 min at room temperature, and permeabilized with 0.5% Triton X-100 for 10 min. After being blocked in 4× saline-sodium citrate (SSC) containing 3% BSA and 0.2% Tween 20 for 30 min at 37°C, the cells were incubated with primary antibodies in detection buffer (4× SSC containing 1% BSA and 0.2% Tween 20) for 1 hr at 37°C, washed twice with 4× SSC, and incubated for 1 hr at 37°C with secondary antibodies conjugated with Alexa Fluor 488 or Alexa Fluor 555. For DNA staining, fixed cells were incubated with 0.2 µg/mL 4′,6-diamidino-2-phenylindole dihydrochloride (DAPI) or 1 µg/mL Hoechst 33342 and then washed in 4× SSC.

The following antibodies were used: anti-Disabled-2/p96 (Dab2) mouse monoclonal antibody (clone 52, BD Biosciences, 610464), anti-Gata4 rabbit polyclonal antibody (Santa Cruz Biotechnology, sc-9053), anti-alpha-SMA mouse monoclonal antibody (clone 1A4, Sigma, A5228), anti-Sox17 polyclonal goat antibody (R&D Systems, AF1924), goat anti-mouse IgG conjugated with Alexa Fluor 488 (Invitrogen, A11017), goat anti-rabbit IgG conjugated with Alexa Fluor 488 or Alexa Fluor 555 (Invitrogen, A11070, A21430), and rabbit anti-goat IgG conjugated with Alexa Fluor 488 (Invitrogen, A21222).

### RNA Expression Analysis by RT-PCR and RT-qPCR

For RT-PCR, total RNA was isolated with TRIzol Reagent (Invitrogen), and the first-strand cDNA was synthesized from 1–5 µg of total RNA with random hexamer primers and SuperScript II reverse transcriptase (Invitrogen), according to the manufacturer's protocol. Primer sequences and PCR conditions are listed in Table S4.

For RT-qPCR, cytoplasmic RNA was isolated with the RNeasy Mini Kit (Qiagen) according to the manufacturer's cytoplasmic RNA protocol with the DNase digestion option. The first-strand cDNA was synthesized from 400 ng of total RNA with SuperScript VILO cDNA synthesis kit (Invitrogen), according to the manufacturer's protocol. Expression levels of genes of interest in cDNA samples were quantitated by real-time PCR using FastStart SYBR Green Master (Roche Applied Science), based on a standard curve using genomic DNA. The RT-qPCR data were normalized by values of three housekeeping genes, *Gapdh*, *Rps21* and *Rps27a* as internal control genes. Primer sequences and PCR conditions are listed in Table S4.

### Microarray and Data Analysis

For the Affymetrix microarray analysis, total RNA was isolated with TRIzol reagent and purified using an RNeasy Mini Column (Qiagen). The cRNA probe was prepared using a two-cycle target-labeling assay and hybridized to Affymetrix MOE430v2 oligonucleotide arrays as recommended by the manufacturer (Affymetrix). RNA from two independent experiments were used as duplicates for each experimental condition. The microarray data were analyzed using the Affy package [76] of the Bioconductor suite of programs [77] in combination with the eXintegrator system [78] (http://www.cdb.riken.jp/scb/documentation/). Data from the raw .CEL files were used either to calculate expression values using RMA expression values or as input to the eXintegrator system. A Principal Component Analysis (PCA) was carried out using the R “prcomp” function [79], and differentially expressed genes were identified using the SAM algorithm [80].

For Agilent microarray analysis, total RNA was isolated with an RNeasy Plus Micro Kit with a gDNA Eliminator column (Qiagen) in most cases. For the short-term Gata4-response experiment (0–3 hr), cytoplasmic RNA was isolated with the RNeasy Mini Kit (Qiagen) according to the manufacturer's cytoplasmic RNA protocol with the DNase digestion option. RNA quality was checked by electrophoresis on an Agilent 2100 Bioanalyzer (Agilent Technologies). Fifty nanograms of total RNA was labeled by a Low Input Quick Amp Labeling Kit (Agilent Technologies) and hybridized to a Mouse Gene Expression 8x60k Microarray (Agilent Technologies) according to the manufacturer's instructions. For 72 hr-time-course analysis, each RNA was labeled in triplicate (Flk1(+) cells at 0 hr and at 12, 24, 36, 48 and 72 hr in the presence of Dex) or in duplicate (others). For 3 hr-time-course analysis, RNA from two independent experiments for WT or DKO cells expressing Gata4GR transgene (WT+Gata4GR and DKO+Gata4GR) were labeled and used as duplicate for each time point, while each RNA from one experiment was labeled in duplicate for DKO cells without the Gata4GR transgene (DKO). The microarray data were quantile-normalized and analyzed using custom R scripts with the Limma package [81], the Bioconductor package suite [77], and custom Perl scripts. Probe values from the same gene were merged into the mean values to calculate the gene expression values, and the analyses described below were performed only for genes that had a GeneSymbol. To identify differentially expressed genes, we used empirical Bayes methods [82]. Genes that had a p value <0.01 and fold change >2 or 4 were selected. Unsupervised hierarchical clustering was performed in the clustering module for Perl [83] with 1 - (Pearson correlation coefficient) as a distance and average linkage. The clusters were visualized by Java Treeview (http://jtreeview.sourceforge.net/) [84] and custom Perl scripts. Venn diagrams were generated using the BioVenn web application (http://www.cmbi.ru.nl/cdd/biovenn/) [85]. Gene ontology analysis at Biological Process level 4 (BP4) was performed using DAVID (http://david.abcc.ncifcrf.gov/) [86] version 6.7 with default parameters.

The microarray data have been deposited into the Gene Expression Omnibus (GEO) database (accession number GSE36814 for the experiment using the type IV collagen-coating condition by Affymetrix microarray analysis and GSE36313 for the experiment using the OP9 co-culture condition by Agilent microarray analysis).

### Chromatin Immunoprecipitation and High-Throughput Sequencing (ChIP-seq) and Data Analysis

ChIP was performed using the ChIP-IT Express chromatin immunoprecipitation kit (Active Motif, Rixensart, Belgium) according to the manufacturer's instructions. Briefly, WT or DKO ES cells differentiated on 15-cm dishes for 4.5 days using OP9-co-culture were treated with Dex for 3 hours and then collected by trypsinization. The differentiated cells were stained with a biotin-conjugated anti-Flk1 antibody (AVAS12, eBioscience) followed by streptavidin microbeads (Miltenyi Biotec), and then sorted with a magnetic cell separation system (MACS, Miltenyi Biotec). The isolated mesoderm cells were crosslinked with 1% formaldehyde for 10 min at room temperature, then the formaldehyde was quenched by adding glycine to a final concentration of 0.125 M. Chromatin was sonicated to an average size of 0.3–0.5 kb using Covaris shearing technology (Covaris, Massachusetts, USA). A mixture of equal amounts of three anti-Gata4 antibodies (sc-1237 and sc-25310, Santa Cruz Biotechnology and L97-56, BD Biosciences), bound to magnetic beads (Active Motif), was added to the sonicated chromatin, and the mixture was incubated for 4 hours at room temperature. After the beads were washed, the chromatin was eluted, and the crosslinking was then reversed. The DNA was purified with a MinElute DNA purification kit (Qiagen). The resultant ChIP DNA was quantified using a Bioanalyzer (Agilent Technologies) and a Quant-it dsDNA assay kit (Invitrogen). Undifferentiated WT or DKO ES cells without Dex treatment were used as controls for ChIP.

Libraries for high-throughput sequencing were prepared with the Illumina ChIP-seq DNA Sample Prep Kit according to the manufacturer's instructions. High-throughput sequencing using an Illumina Hiseq and mapping of the resulting reads were performed by Hokkaido System Science Co., Ltd. Japan. Data analysis and visualization of the sequence reads were performed with DNAnexus software tools (https://dnanexus.com/). The ChIP-seq raw data have been deposited into the GEO database (accession code GSE41361). Of the initial 250,590,286 reads for WT Flk1+ cells and 392,760,766 reads for DKO Flk1+ cells obtained in the Gata4-ChIP-seq experiment, 209,514,577 (83.61%) and 368,177,514 (93.74%) were mapped to the mouse reference genome (NCBI v37, mm9), respectively.

The ChIP-seq peaks for Gata4 were determined with DNAnexus software tools using the following settings: KDE (kernel density estimation) bandwidth = 30, ChIP candidate threshold = 5.0, Experiment to background enrichment = 3.0, Minimum ratio of confident to repetitive mapping in region = 3.0. Peak calling with DNAnexus software tools identified 20,410 peaks for WT Flk1+ cells and 22,733 peaks for DKO Flk1+ cells using WT or DKO ES ChIP-seq reads, respectively, as background controls. For DKO-specific Gata4 peak calling, 10,636-enriched peaks were identified from the comparison between WT and DKO Flk1+ cells. The nearest Refseq genes (within 5 kb) were identified from the Gata4 peaks. Transcription-factor binding-site motif analysis for the Gata4 peak sequences was performed using the MEME-ChIP suit (http://meme.nbcr.net/) [50].

### ChIP-qPCR Analysis

For ChIP-qPCR, 100 nM Dex was added to WT ES cells stably expressing Gata4GR cultured on 15-cm dishes in ES medium containing 10% FCS. At 3 hours after the addition of Dex, the cells were crosslinked with 1% formaldehyde for 10 min at room temperature on the dishes; then the formaldehyde was quenched by adding glycine to a final concentration of 0.125 M. The fixed cells were recovered by scraping with a rubber policeman in ice-cold PBS and collected by centrifugation (Dex+). WT ES cells without Dex treatment were used as controls (Dex−). Chromatin sonication, ChIP with the mixture of three anti-Gata4 antibodies, and purification of ChIP DNA were performed as described for ChIP-seq, except for using Protein G FG beads (Tamagawa seiki) instead of magnetic beads in the kit. Relative abundance of regions of interest in precipitated DNA to input DNA was quantitaed by real-time PCR using Thunderbird SYBR qPCR Mix (TOYOBO) with the comparative CT method. Gata4 enrichment was calculated as fold change of relative abundance for Dex+ to that for Dex−. Primer sequences and PCR conditions are listed in Table S4.

### DNA Methylation Analysis by Bisulfite Sequencing

Genomic DNA was isolated from ES cells or Flk1(+) mesoderm cells with a QIAamp DNA micro kit (Qiagen) and subjected to bisulfite conversion with an EpiTect Bisulfite kit (Qiagen), according to the manufacturer's instructions with a slight modification [87]. Target sequences of the bisulfite-converted DNA were amplified by PCR, and 24 clones for each sample were sequenced. The primers for bisulfite sequencing were designed using MethPrimer (http://www.urogene.org/methprimer/) [88]. The bisulfite sequencing data were analyzed using QUMA (http://quma.cdb.riken.jp/) [89]. Primer sequences and PCR conditions are listed in Table S4.

### Luciferase Reporter Assay

Constructs used for the luciferase reporter assays of Gata4-ChIP target sequences were based on the pFgf3_1.7k-luc vector [39], in which a fragment of DNA encompassing 1.7 kb of sequence immediately 5′ of the *Fgf3* coding region containing GATA binding sites [90] was inserted into pGL4.10 (Promega). We generated the pFGF3_0.8k-luc vector by removing the 5′-half (0.9 kb) of FGF3 1.7 kb fragment from the pFgf3_1.7k-luc vector with digestion of 5′-end XhoI site on GL4.10 vector and internal AflII site. Luciferase reporter vectors containing Gata4-ChIP target sequences were constructed by inserting a 0.2–0.3 kb fragments centered around Gata4-ChIP-seq peaks amplified with HotStarTaq DNA polymerase (Qiagen) using primers containing XhoI site (forward) and AflII site (reverse) sites, between the XhoI and AflII sites of the pFgf3_0.8k vector (See also Figure S12A). The following Gata4-ChIP peak regions were used for the construction and luciferase reporter assay; *Aqp8* (intron), *Grk5* (promoter), *Sord* (promoter), *Sox7* (promoter), *Lgmn* (intron), *Myocd* (intron), and *Spon1* (3′UTR). Primer sequences are listed in Table S4.

For transfection of reporter plasmids, 1×10^4^ cells were seeded in each well of a 96-well plate in ES medium containing 10% FCS, and incubated with 330 ng reporter plasmid and 8 ng of the internal control plasmid pRL-CMV (Promega), together with Lipofectamine 2000 (Invitrogen), following the manufacturer's protocol. At 3 hr after the transfection, 100 nM Dex was added. Luciferase assays were performed at 30 hr after the addition of Dex using a Dual-luciferase assay kit (Promega).

## Supporting Information

Figure S1 Suppression of Gata4-induced primitive endoderm differentiation during leukemia inhibitory factor (LIF) withdrawal-induced ES-cell differentiation. (*A*) Experimental conditions for Gata4-induced primitive endoderm differentiation. Wild-type (WT) ES cells stably expressing Gata4 fused with the ligand-binding domain of human glucocorticoid receptor (Gata4GR) were established. Gata4GR in these ES cells was activated by adding dexamethasone (Dex), a glucocorticoid receptor ligand, to cells cultured under either the undifferentiated (LIF(+)) or differentiated (LIF(−)) condition. For the LIF(−) condition, Dex was added to the cultures 3 days after the withdrawal of LIF to activate Gata4GR. (*B*) Morphology of the differentiated ES cells in response to Gata4GR activation under the LIF(+) or LIF(−) condition. (*C*) Expression profile of differentiation marker genes in WT ES cells in response to Gata4GR activation under the LIF(+) or LIF(−) condition. Total RNA was isolated at the time points shown in (*A*) and analyzed by RT-PCR for primitive endoderm markers (*Gata4*, *Gata6*, *Sox7*, *Dab2*, *Afp*, *Hnf4a*, *Foxa2*, and *Fgf3*), mesoderm markers (*Brachyury* and *Bmp4*), and a neuroectoderm marker (*Isl1*). *Gapdh* was the loading control. *Gata4GR represents the transgene transcript for Gata4GR. The Gata4 primer set amplified the endogenous transcript but not the Gata4GR transcript. The number of PCR cycles is shown at the right. (*D*) Immunofluorescence analysis of Disabled2 (Dab2) and Gata4 in the wild-type ES cells before and after Gata4GR activation under the LIF(+) or LIF(−) condition. The cells were stained with anti-Dab2 (green) and anti-Gata4 (red) antibodies and counterstained with DAPI (blue). In the LIF(+) condition, Gata4GR activation caused strong expression of the primitive endoderm markers Dab2 and Gata4 (LIF(+) Dex+, day 4). In the LIF(−) condition, Gata4GR activation did not lead to Dab2 expression in the wild-type cells, although moderate Gata4 staining was detected in their nuclei (LIF(−) Dex+, day 3). Note that the anti-Gata4 antibody detected both the endogenous Gata4 and exogenous Gata4GR proteins. Undifferentiated ES cells grew as densely packed colonies (LIF(+), day 0).
(TIF)

Figure S2 Phase-contrast photomicrographs of primitive endoderm cells directly differentiated from WT or *Dnmt3a* ^−/−^ *Dnmt3b* ^−/−^ (DKO) ES cells. WT or DKO ES cells expressing Gata4GR were cultured for 4 days with Dex in the presence of LIF (ES, Dex+).
(TIF)

Figure S3 Flow cytometry profiles and immunofluorescence analysis of differentiated *Dnmt1* ^−/−^ Flk1(+) cells. (*A*) Flow cytometry profiles of Flk1 and E-cadherin in differentiated *Dnmt1* ^−/−^ ES cells expressing Gata4GR (1KO) using the OP9 co-culture method. The percentage of Flk1(+)/E-cadherin(−) cells is indicated. (*B, C*) Immunofluorescence analysis of alpha-smooth muscle actin (SMA) (*B*) and Dab2 (*C*) in Flk1(+) mesoderm cells that were derived from differentiated *Dnmt1* ^−/−^ ES cells expressing Gata4GR (1KO) using the OP9 co-culture method and were cultured for 4 days with or without Dex. SMA and Dab2, green; Hoechst 33342, blue. Scale bar, 50 µm. These experiments were performed twice.
(TIF)

Figure S4 Gata4-induced primitive endoderm differentiation from DKO Flk1(+) cells derived under type IV collagen culture. (*A*) Experimental strategy for isolating mesoderm progenitors from ES cells using type IV collagen culture conditions and the subsequent activation of Gata4. WT or DKO ES cells stably expressing Gata4GR were differentiated on type IV collagen for 4 days. The Flk1(+) mesoderm cells (Me) were sorted and cultured on type IV collagen with or without Dex to activate Gata4GR. (*B*) Flow cytometry profiles of Flk1 and PDGFRα in differentiated ES cells. WT or DKO ES cells were cultured in type IV collagen-coated dishes for 3, 4, or 5 days and analyzed by flow cytometry using anti-Flk1 and -PDGFRα antibodies. The percentage of cells in each quadrant is indicated. (*C*) Morphology of cells differentiated from Flk1(+) cells with or without Dex. (*D*) Immunofluorescence analysis of differentiation markers in Flk1(+)-derived cells stained with an anti-SMA or anti-Dab2 antibody (green). DNA was stained with DAPI (blue). (*E*) RT-PCR expression analysis of mesoderm (Me) and primitive endoderm (PE) markers in Flk1(+) mesoderm cells cultured for 4 days in the presence or absence of Dex. *Gata4GR, transgene transcript for Gata4GR, not amplified by the Gata4 primer set. (*F*) Expression profiles of ES-cell-derived mesoderm and primitive endoderm cells by DNA microarray analysis. Principal component analysis using a subset of 3,235 probe sets selected on the basis of their internal probe pair co-variances. WT (black) or DKO (red) ES cells carrying Gata4GR were differentiated as described in (*A*). ES, undifferentiated ES cells; PE, primitive endoderm cells derived directly from ES cells; Flk1+, Flk1(+)/PDGFRα(+) mesoderm cells; Dex− and Dex+, Flk1(+)/PDGFRα(+) cells cultured for 4 days with and without Dex, respectively. Note that for both WT and DKO cells, one of the Flk1+ populations shown was derived from parental ES cells not carrying the Gata4GR transgene, so there was more variation in the Flk1+ cells' plotted positions. (*G*) Gene expression profiles extracted from DNA microarray data. ES, pluripotency-associated genes; PE, primitive endoderm genes; Me, mesoderm genes; SM, smooth muscle genes. Expression values are represented as colors, from lowest (red) to highest (blue). Efficient Gata4-induced primitive endoderm differentiation from DKO Flk1(+) cells derived under type IV collagen culture were observed at least in five experiments shown by morphologies, immunofluorescence, RT-PCR or microarray analysis.
(TIF)

Figure S5 Extraction of Gata4-hyper-responsive genes at 24 hr in Dnmt3a/Dnmt3b-deficient Flk1(+) mesoderm cells from transcriptome data. Venn diagram of the 2-fold upregulated genes in DKO mesoderm with Gata4 activation at 24 hr compared to (i) WT cells under the same conditions (WT Dex+<DKO Dex+, purple), (ii) the same cells without Gata4 activation (DKO Dex−<DKO Dex+, light green), or (iii) the same cells before Gata4 activation (DKO 0 h<DKO Dex+, orange). The overlapping genes of these three categories (146 genes, marked with an asterisk) are considered genes that are upregulated in response to Gata4 preferentially at low DNA methylation levels.
(TIF)

Figure S6 Heat map of temporal transcriptional profiles for 320 Gata4-responsive genes. WT or DKO Flk1(+) mesoderm cells or ES cells expressing Gata4GR were cultured for 72 hr in the presence or absence of Dex, and expression microarray data were obtained at several time points (0, 12, 24, 36, 48, and 72 hr for Flk1(+) mesoderm cells; 0, 3, 6, 12, 24, 48, and 72 hr for ES cells). The 320 genes that responded more to Gata4 in DKO than WT Flk1(+) mesoderm cells at 72 hr were extracted as described in Figure 2D. Clustering of these 320 genes was based on their temporal expression profiles in Flk1(+) mesoderm and ES cells, and the resulting dendrogram is shown at the left. Relative gene expression values (log2) are represented as colors, from lowest (blue) to highest (yellow). The maximum, mean, and minimum of all gene expression values (log2) in these experimental samples are also shown at the right. Genes used in Figure 3 and Figure S7 are highlighted as blue circles or red triangles.
(TIF)

Figure S7 Temporal expression changes of individual Gata4-hyper-responsive genes in Flk1(+) mesoderm cells and ES cells. The mean values of triplicates (Flk1+, 0 hr and Dex+) or duplicates (others) from the microarray data with their standard deviations are shown. (*A*–*C*) Group 1 genes responded to Gata4 in WT and DKO ES cells. (*A*) Genes expressed in endoderm-derived tissues. (*B*) Genes whose expression was not restricted to endoderm-derived tissues. (*C*) Primitive endoderm genes. (*D*) Primitive endoderm genes that were also expressed in Flk1(+) mesoderm cells. (*E*) Primitive endoderm genes that did not respond to Gata4 in DKO Flk1(+) mesoderm cells. Note that smaller scales for the expression signal are used for Flk1(+) mesoderm cells compared to those for ES cells to present temporal expression changes in (*A* to *E*). (*F*–*H*) Group 2 genes did not respond to Gata4 in ES cells. (*F*) Cardiac genes. (*G*) Genes expressed in mesoderm-derived tissues. (*H*) Genes expressed in endoderm-derived tissues. “WT+Gata4GR Dex+” and “WT+Gata4GR Dex−”, WT cells expressing Gata4GR with and without Dex, respectively; “DKO+Gata4GR Dex+” and “DKO+Gata4GR Dex−”, DKO cells expressing Gata4GR with and without Dex, respectively.
(TIF)

Figure S8 RT-qPCR analysis of Gata4 response genes in Flk1(+) mesoderm cells. ES cells differentiated on OP9 stromal cells were treated with Dex for 0, 1, 2 or 3 hr. The Flk1(+) mesoderm cells were then sorted by flow cytometry, and their gene expression was analyzed using RT-qPCR. The mean values of triplicates for gene expression levels with their standard deviations are shown. “Exp. 1” and “Exp. 2” represent results of independent experiments performed separately. “WT+Gata4GR Dex+”, WT cells expressing Gata4GR with Dex; “DKO+Gata4GR Dex+”, DKO cells expressing Gata4GR with Dex; “DKO Dex+”, DKO cells without the Gata4GR transgene with Dex.
(TIF)

Figure S9 Central motif-enrichment analysis for Gata4-ChIP-seq peaks of WT or DKO Flk1(+) cells with Gata4 activation. ChIP-seq peak regions centered within a 500-bp region for WT (20,410 peaks) or DKO (22,733 peaks) were used as the input for CentriMo. Transcription-factor-binding motifs consisting of vertebrate motifs in the JASPAR CORE database and motifs for mouse transcription factors in the UniPROBE database were used for the motif enrichment analysis. The site-probability curves (A) and the statistics and values (B) for the three most highly ‘centrally enriched’ motifs for WT or DKO are shown. The shape of the site-probability curves and the number of sequences for which the best match to the motif fell in the central region (Bin sites) compared to the number of sequences containing a match to the motif (Total sites) indicated that the Gata motifs were highly centrally enriched in both the WT and DKO Gata4-ChIP-seq peaks.
(TIF)

Figure S10 Gata4 ChIP-qPCR analysis at *Aqp8* locus for WT ES cells with Gata4 activation. (*A*) Relative abundance of regions of interest in Gata4 ChIP DNA to input DNA was quantitated by qPCR. Fold enrichment of Gata4 was calculated as the ratio of relative abundance of ChIP DNA in the presence of Dex versus that in the absence of Dex (Dex+/−). (*B*) The mapped read enrichment from Gata4 ChIP-seq experiments in WT and DKO Flk1(+) cells at *Aqp8* locus, an enlarged part of Figure 5B, is shown. The primer-set positions for ChIP-qPCR (T1, C1, C2, T3 and T4) are shown as horizontal bars.
(TIF)

Figure S11 Gata4-binding-site profiles, DNA-methylation states, and Gata4-induced temporal expression changes of Gata4-response genes and neighboring genes. Gata4 ChIP-seq enrichment at the (*A*) *Lgmn*, (*B*) *Mrap*, and (*C*) *Thbs4* loci in WT or DKO Flk1(+) cells in which Gata4GR was activated by Dex addition is shown. Tracks represent the mapped read enrichment as determined by DNAnexus software. Blue arrowheads mark Gata4 peaks enriched in DKO Flk1(+) cells compared to WT Flk1(+) cells. DNA methylation states at transcription start sites and Gata4-binding sites were analyzed by bisulfite sequencing. Horizontal bars represent the genomic regions subjected to DNA methylation analysis. Line graphs show the temporal expression changes for the indicated genes from microarray data at several time points within 72 hr in WT or DKO Flk1(+) mesoderm cells and ES cells in the presence or absence of Dex. (*A*) *Lgmn* and its neighboring gene *Rin3* were associated with Gata4 peaks enriched in DKO Flk1(+) cells compared to WT Flk1(+) cells. *Rin3* itself did not transcriptionally respond to Gata4, suggesting that the Gata4 peak located in the 3′ region of *Rin3* contributes to the *Lgmn* transcription. Both Gata4 peak regions were methylated in a Dnmt3-dependent manner, and the peak region at *Rin3* was *de novo* methylated during mesoderm commitment. (*B*) The high-CpG promoter of *Mrap* was heavily methylated in a Dnmt3-dependent manner. Although *Mrap* immediately responded to Gata4 in DKO mesoderm cells, no appreciable Gata4 peaks were associated with its proximal genomic region. One Gata4 peak was observed in the neighboring gene, *2610039C10Rik*, in both WT and DKO mesoderm cells, while *2610039C10Rik* itself did not respond to Gata4. (*C*) *Thbs4* was associated with Gata4 binding at the intronic region in both WT and DKO mesoderm cells, and its promoter region was *de novo* methylated during mesoderm differentiation.
(TIF)

Figure S12 Gata4-dependent enhancer activity of DNA fragments associated with Gata4 ChIP-seq peaks. (*A*) Schematic diagrams of Gata4-dependent reporter constructs used for luciferase reporter assay. pFGF3_1.7k, Luciferase reporter plasmid containing a 1.7 kb *Fgf3* fragment including both Gata4-binding sites and promoter (P). pFGF3_0.8k, Luciferase reporter plasmid containing the 0.8 kb *Fgf3* promoter only. ChIP target fragments (0.2–0.3 kb) associated with Gata4 ChIP-seq peaks (T) were inserted to 5′ of the pFGF3_0.8k promoter at the AflII site (Af). (*B*) Luciferase activity of reporter plasmids containing ChIP-seq peak-associated fragment in response to Gata4 activation by addition of Dex for 30 hr in WT ES cells. Fold changes in luciferase activities in the presence versus the absence of Dex (Dex+/−) were calculated for each reporter construct. The means of triplicates with the standard deviations are shown. pFGF3_1.7k (pFGF3) and pFGF3_0.8k (empty) were used as positive and negative controls, respectively. Asterisks represent statistically significant differences (P<0.05, Student's t-test) compared to the negative control (empty).
(TIF)

Table S1 The 20 most significantly enriched gene ontology terms among the differentially expressed genes in WT or Dnmt3a/Dnmt3b-deficient ES or Flk1(+) mesoderm cells with or without Gata4 activation, as categorized in Figure 2B.
(PDF)

Table S2 The 20 most significantly enriched gene ontology terms among the upregulated genes in Dnmt3a/Dnmt3b-deficient Flk1(+) mesoderm cells with Gata4 activation, as categorized in Figure 2C.
(PDF)

Table S3 The List of Gata4 hyper-responsive genes at 24 hr in Dnmt3a/Dnmt3b-deficient mesoderm cells, as categorized in Figure 3A.
(XLS)

Table S4 Primers and PCR conditions used in this study.
(PDF)
