# Supplementary figures and images for: DNA Methylation Restricts Lineage-specific Functions of Transcription Factor Gata4 during Embryonic Stem Cell Differentiation
Source: PLoS Genet. 2013 Jun 27;9(6):e1003574. doi: 10.1371/journal.pgen.1003574 (PMC3694845; doi:10.1371/journal.pgen.1003574)

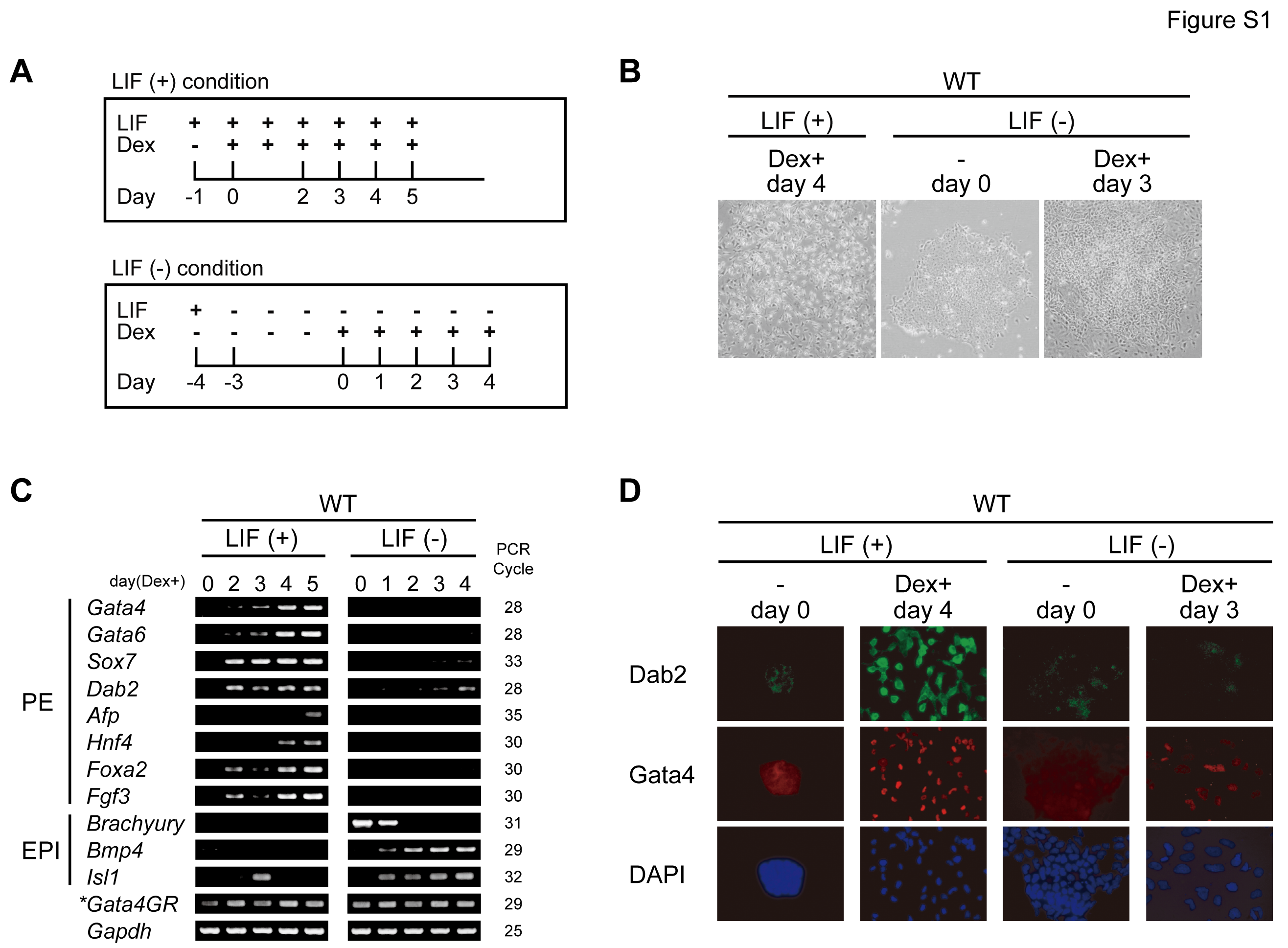

Supplement: Figure S1 — Suppression of Gata4-induced primitive endoderm differentiation during leukemia inhibitory factor (LIF) withdrawal-induced ES-cell differentiation. (A) Experimental conditions for Gata4-induced primitive endoderm differentiation. Wild-type (WT) ES cells stably expressing Gata4 fused with the ligand-binding domain of human glucocorticoid receptor (Gata4GR) were established. Gata4GR in these ES cells was activated by adding dexamethasone (Dex), a glucocorticoid receptor ligand, to cells cultured under either the undifferentiated (LIF(+)) or differentiated (LIF(−)) condition. For the LIF(−) condition, Dex was added to the cultures 3 days after the withdrawal of LIF to activate Gata4GR. (B) Morphology of the differentiated ES cells in response to Gata4GR activation under the LIF(+) or LIF(−) condition. (C) Expression profile of differentiation marker genes in WT ES cells in response to Gata4GR activation under the LIF(+) or LIF(−) condition. Total RNA was isolated at the time points shown in (A) and analyzed by RT-PCR for primitive endoderm markers (Gata4, Gata6, Sox7, Dab2, Afp, Hnf4a, Foxa2, and Fgf3), mesoderm markers (Brachyury and Bmp4), and a neuroectoderm marker (Isl1). Gapdh was the loading control. *Gata4GR represents the transgene transcript for Gata4GR. The Gata4 primer set amplified the endogenous transcript but not the Gata4GR transcript. The number of PCR cycles is shown at the right. (D) Immunofluorescence analysis of Disabled2 (Dab2) and Gata4 in the wild-type ES cells before and after Gata4GR activation under the LIF(+) or LIF(−) condition. The cells were stained with anti-Dab2 (green) and anti-Gata4 (red) antibodies and counterstained with DAPI (blue). In the LIF(+) condition, Gata4GR activation caused strong expression of the primitive endoderm markers Dab2 and Gata4 (LIF(+) Dex+, day 4). In the LIF(−) condition, Gata4GR activation did not lead to Dab2 expression in the wild-type cells, although moderate Gata4 staining was detected in their nuclei (LI [file pgen.1003574.s001.tif]

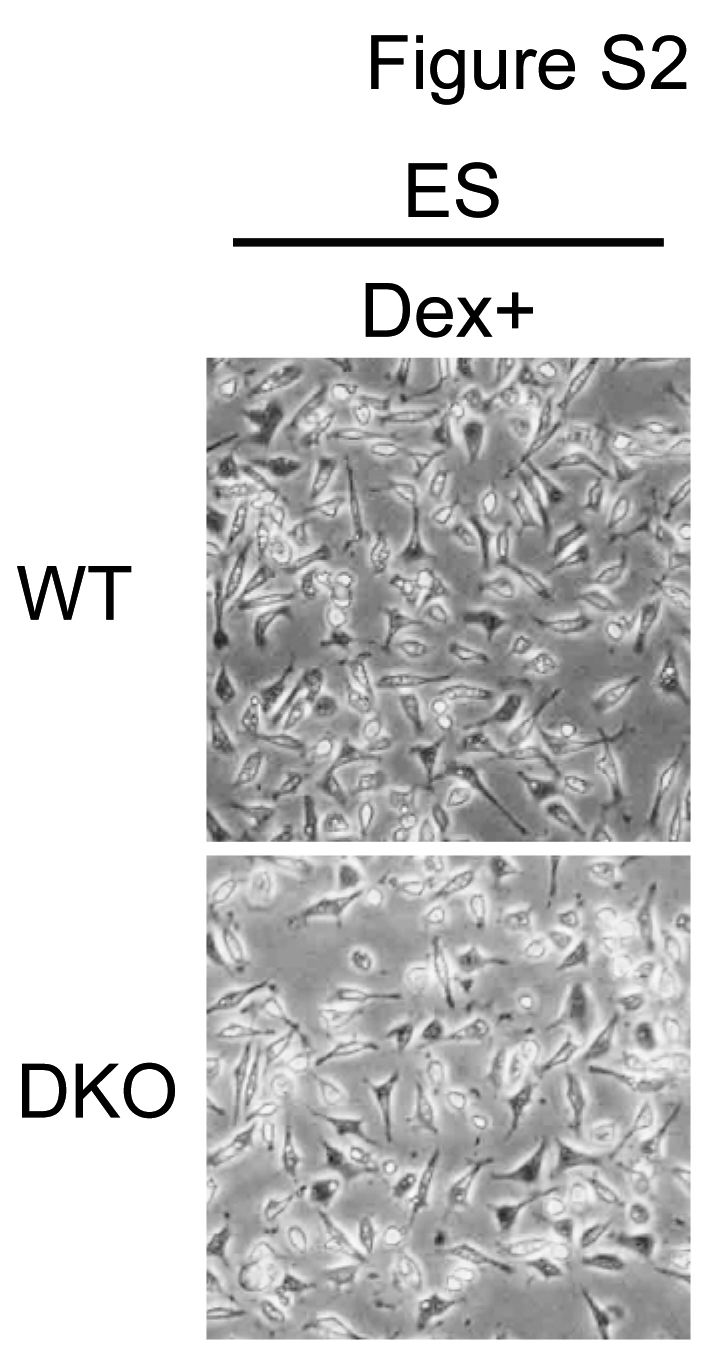

Supplement: Figure S2 — Phase-contrast photomicrographs of primitive endoderm cells directly differentiated from WT or Dnmt3a −/− Dnmt3b −/− (DKO) ES cells. WT or DKO ES cells expressing Gata4GR were cultured for 4 days with Dex in the presence of LIF (ES, Dex+). (TIF) [file pgen.1003574.s002.tif]

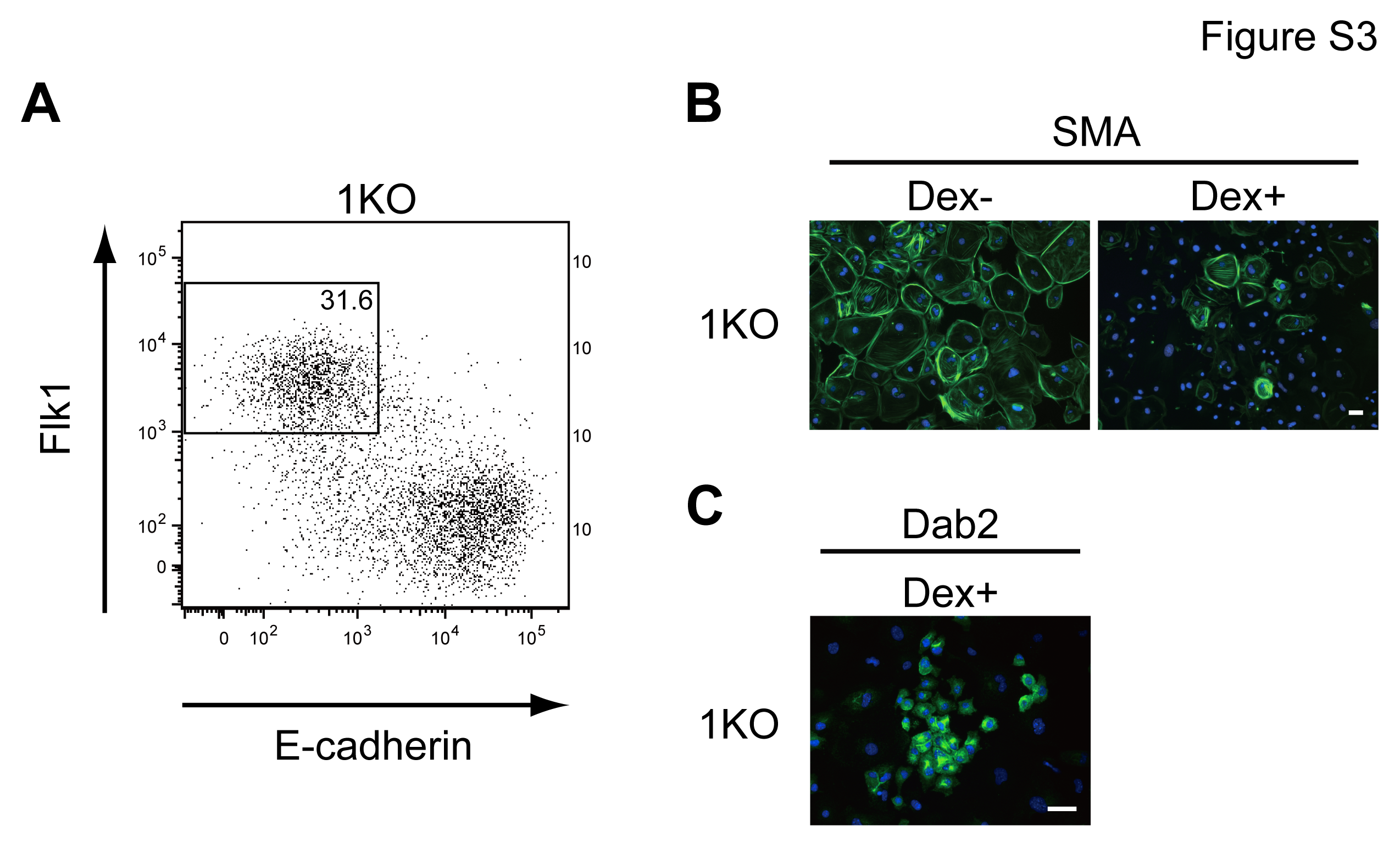

Supplement: Figure S3 — Flow cytometry profiles and immunofluorescence analysis of differentiated Dnmt1 −/− Flk1(+) cells. (A) Flow cytometry profiles of Flk1 and E-cadherin in differentiated Dnmt1 −/− ES cells expressing Gata4GR (1KO) using the OP9 co-culture method. The percentage of Flk1(+)/E-cadherin(−) cells is indicated. (B, C) Immunofluorescence analysis of alpha-smooth muscle actin (SMA) (B) and Dab2 (C) in Flk1(+) mesoderm cells that were derived from differentiated Dnmt1 −/− ES cells expressing Gata4GR (1KO) using the OP9 co-culture method and were cultured for 4 days with or without Dex. SMA and Dab2, green; Hoechst 33342, blue. Scale bar, 50 µm. These experiments were performed twice. (TIF) [file pgen.1003574.s003.tif]

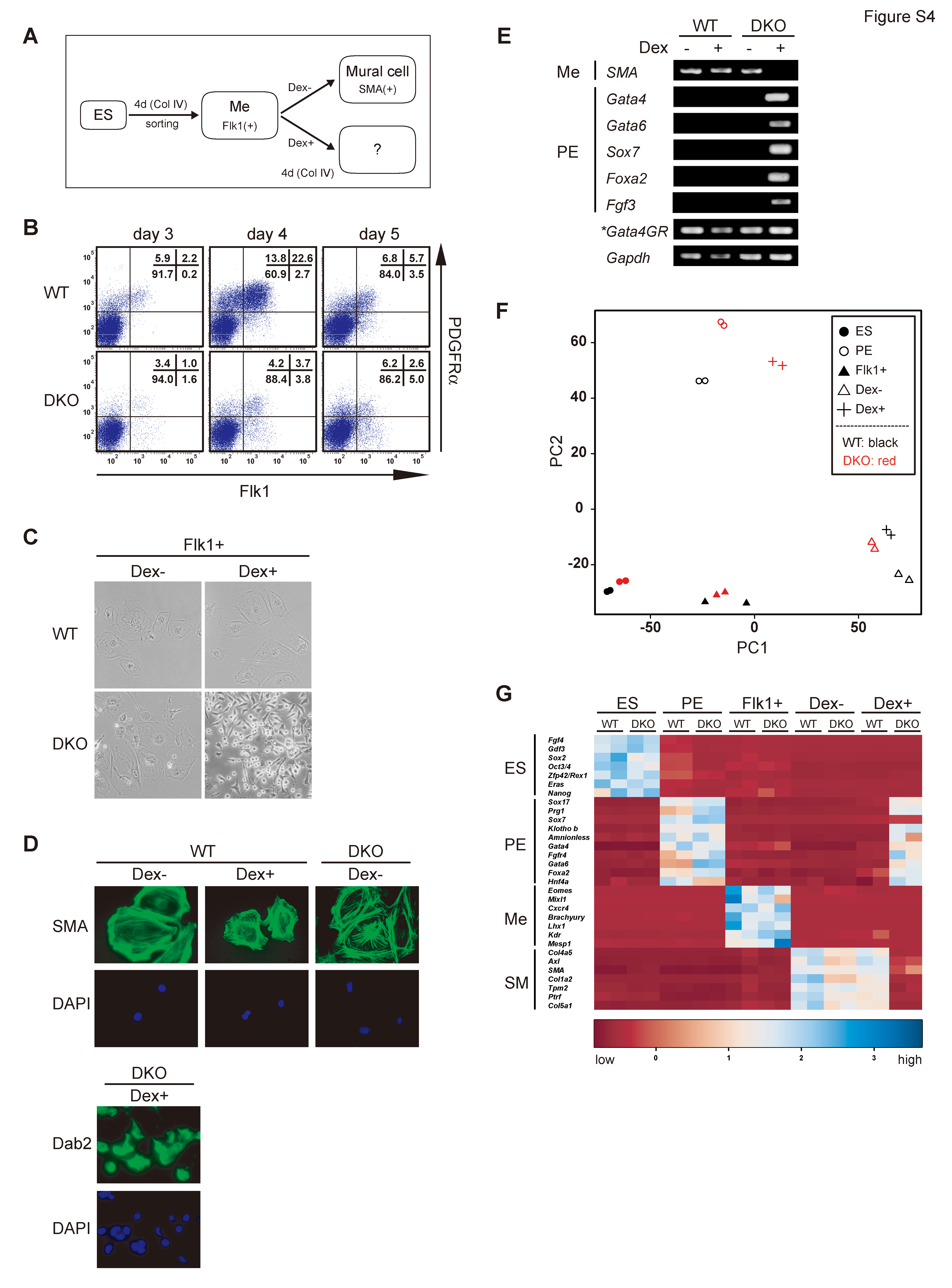

Supplement: Figure S4 — Gata4-induced primitive endoderm differentiation from DKO Flk1(+) cells derived under type IV collagen culture. (A) Experimental strategy for isolating mesoderm progenitors from ES cells using type IV collagen culture conditions and the subsequent activation of Gata4. WT or DKO ES cells stably expressing Gata4GR were differentiated on type IV collagen for 4 days. The Flk1(+) mesoderm cells (Me) were sorted and cultured on type IV collagen with or without Dex to activate Gata4GR. (B) Flow cytometry profiles of Flk1 and PDGFRα in differentiated ES cells. WT or DKO ES cells were cultured in type IV collagen-coated dishes for 3, 4, or 5 days and analyzed by flow cytometry using anti-Flk1 and -PDGFRα antibodies. The percentage of cells in each quadrant is indicated. (C) Morphology of cells differentiated from Flk1(+) cells with or without Dex. (D) Immunofluorescence analysis of differentiation markers in Flk1(+)-derived cells stained with an anti-SMA or anti-Dab2 antibody (green). DNA was stained with DAPI (blue). (E) RT-PCR expression analysis of mesoderm (Me) and primitive endoderm (PE) markers in Flk1(+) mesoderm cells cultured for 4 days in the presence or absence of Dex. *Gata4GR, transgene transcript for Gata4GR, not amplified by the Gata4 primer set. (F) Expression profiles of ES-cell-derived mesoderm and primitive endoderm cells by DNA microarray analysis. Principal component analysis using a subset of 3,235 probe sets selected on the basis of their internal probe pair co-variances. WT (black) or DKO (red) ES cells carrying Gata4GR were differentiated as described in (A). ES, undifferentiated ES cells; PE, primitive endoderm cells derived directly from ES cells; Flk1+, Flk1(+)/PDGFRα(+) mesoderm cells; Dex− and Dex+, Flk1(+)/PDGFRα(+) cells cultured for 4 days with and without Dex, respectively. Note that for both WT and DKO cells, one of the Flk1+ populations shown was derived from parental ES cells not carrying the Gata4GR transgene, so there was more variation [file pgen.1003574.s004.tif]

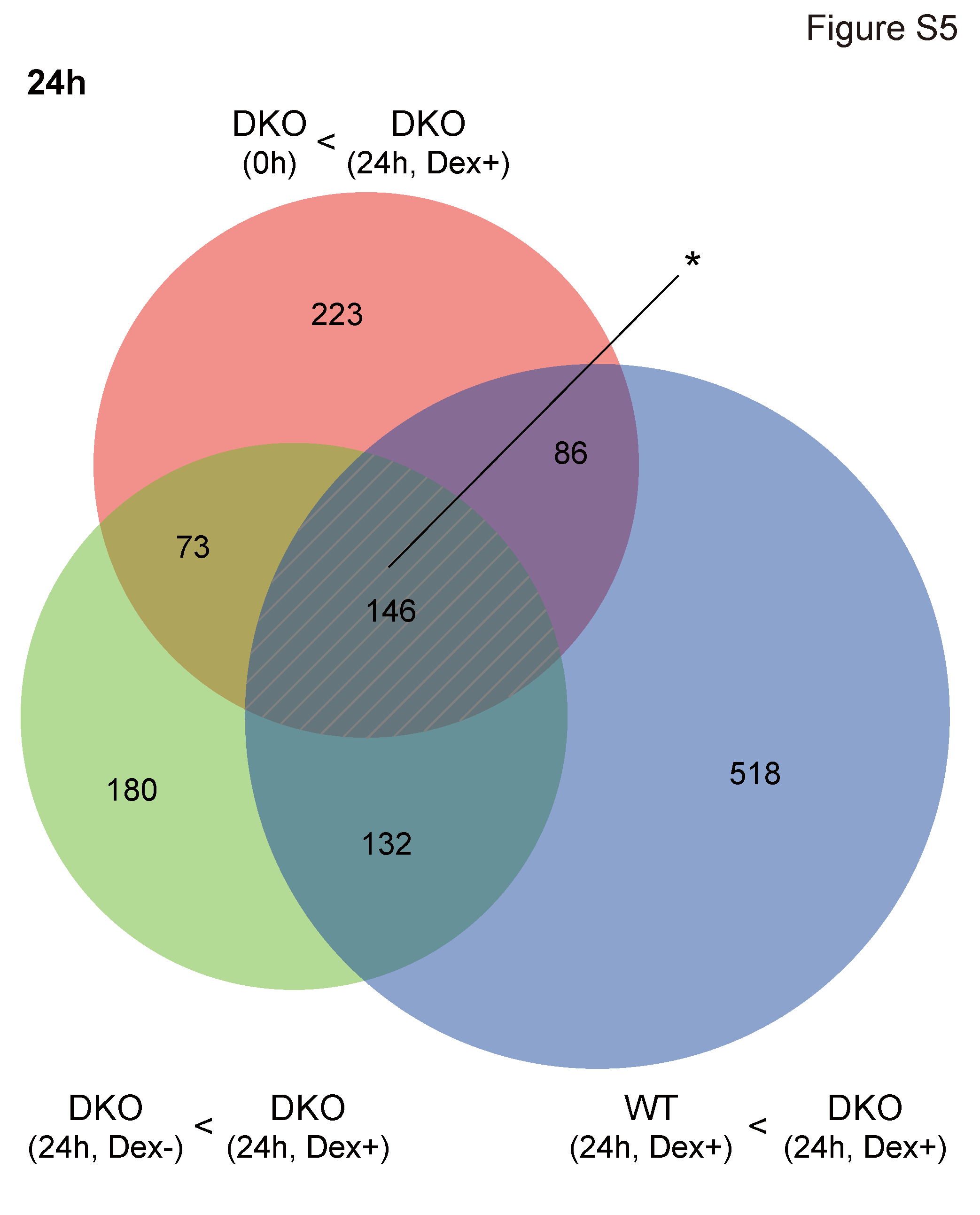

Supplement: Figure S5 — Extraction of Gata4-hyper-responsive genes at 24 hr in Dnmt3a/Dnmt3b-deficient Flk1(+) mesoderm cells from transcriptome data. Venn diagram of the 2-fold upregulated genes in DKO mesoderm with Gata4 activation at 24 hr compared to (i) WT cells under the same conditions (WT Dex+<DKO Dex+, purple), (ii) the same cells without Gata4 activation (DKO Dex−<DKO Dex+, light green), or (iii) the same cells before Gata4 activation (DKO 0 h<DKO Dex+, orange). The overlapping genes of these three categories (146 genes, marked with an asterisk) are considered genes that are upregulated in response to Gata4 preferentially at low DNA methylation levels. (TIF) [file pgen.1003574.s005.tif]

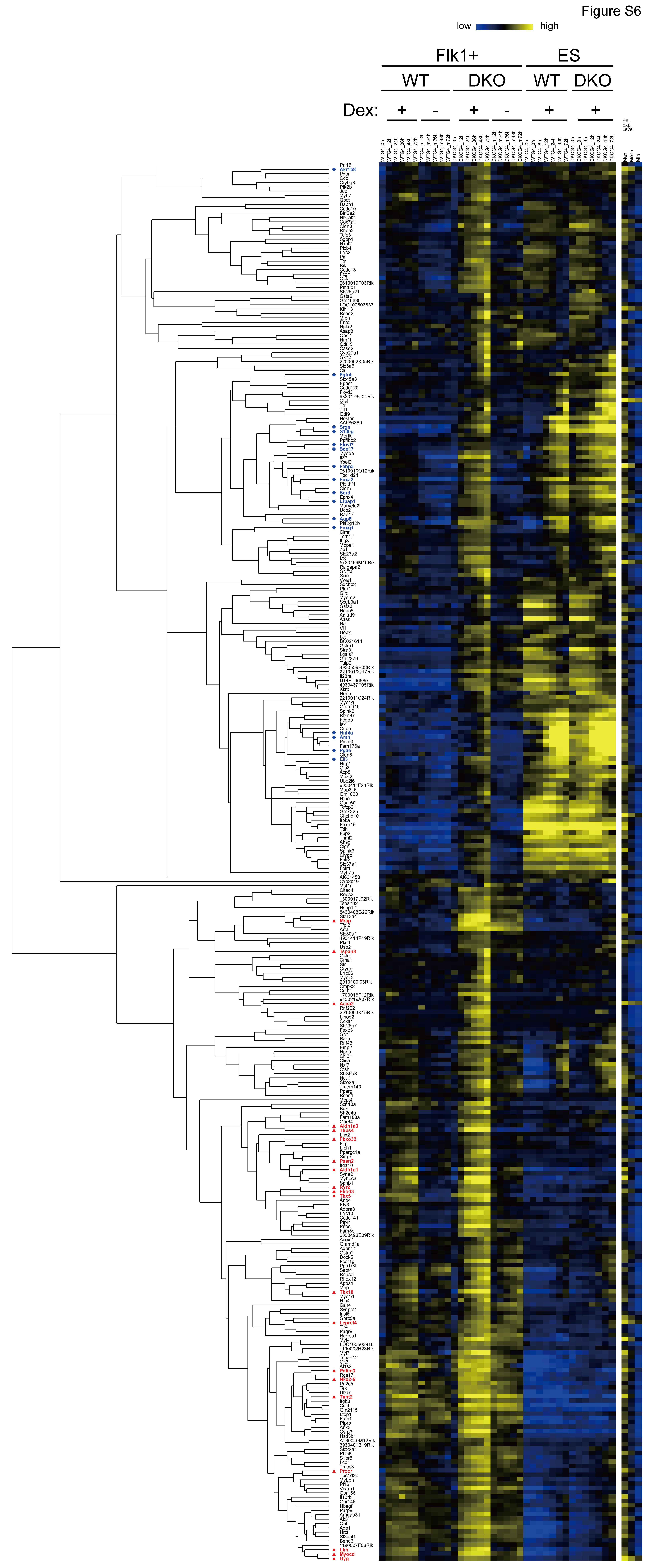

Supplement: Figure S6 — Heat map of temporal transcriptional profiles for 320 Gata4-responsive genes. WT or DKO Flk1(+) mesoderm cells or ES cells expressing Gata4GR were cultured for 72 hr in the presence or absence of Dex, and expression microarray data were obtained at several time points (0, 12, 24, 36, 48, and 72 hr for Flk1(+) mesoderm cells; 0, 3, 6, 12, 24, 48, and 72 hr for ES cells). The 320 genes that responded more to Gata4 in DKO than WT Flk1(+) mesoderm cells at 72 hr were extracted as described in Figure 2D. Clustering of these 320 genes was based on their temporal expression profiles in Flk1(+) mesoderm and ES cells, and the resulting dendrogram is shown at the left. Relative gene expression values (log2) are represented as colors, from lowest (blue) to highest (yellow). The maximum, mean, and minimum of all gene expression values (log2) in these experimental samples are also shown at the right. Genes used in Figure 3 and Figure S7 are highlighted as blue circles or red triangles. (TIF) [file pgen.1003574.s006.tif]

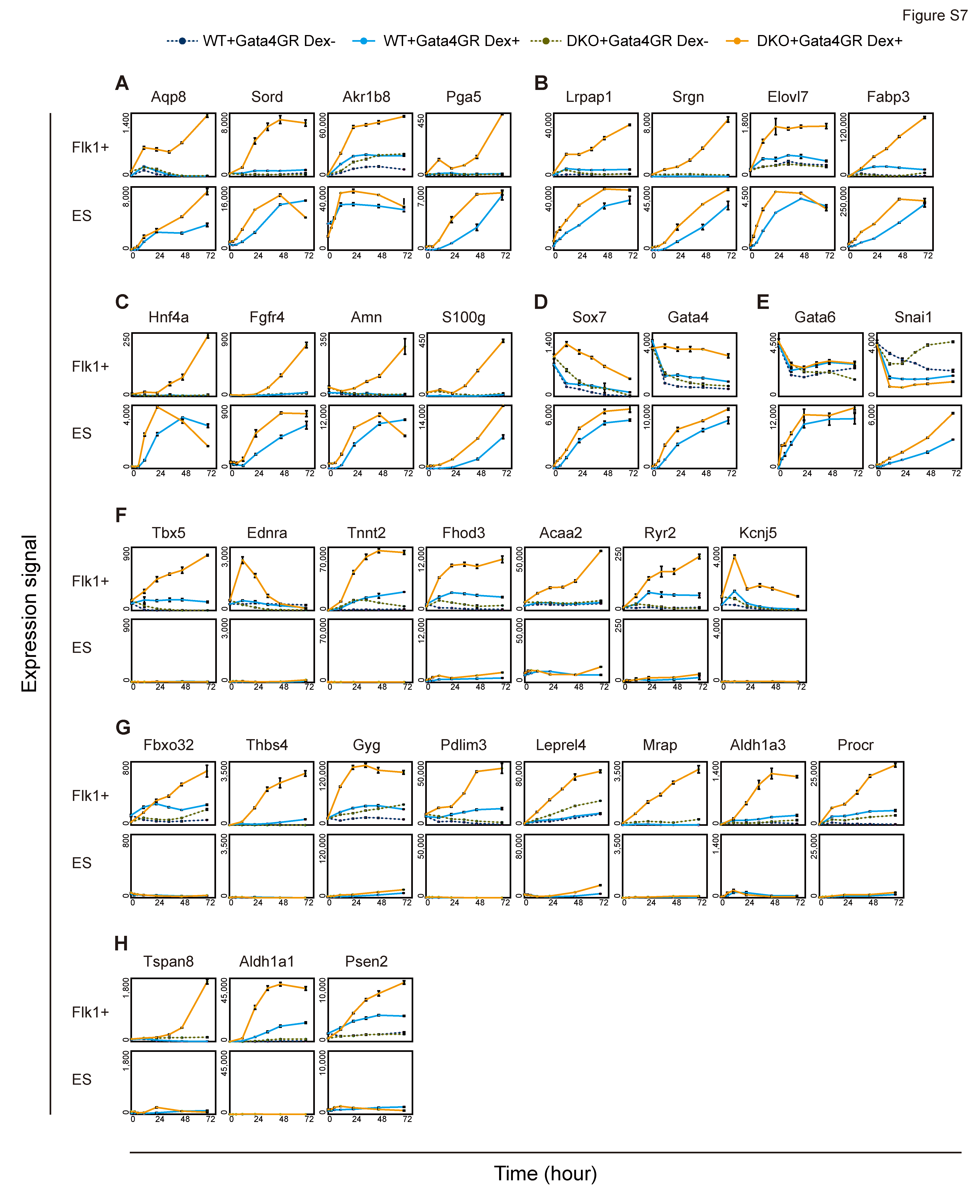

Supplement: Figure S7 — Temporal expression changes of individual Gata4-hyper-responsive genes in Flk1(+) mesoderm cells and ES cells. The mean values of triplicates (Flk1+, 0 hr and Dex+) or duplicates (others) from the microarray data with their standard deviations are shown. (A–C) Group 1 genes responded to Gata4 in WT and DKO ES cells. (A) Genes expressed in endoderm-derived tissues. (B) Genes whose expression was not restricted to endoderm-derived tissues. (C) Primitive endoderm genes. (D) Primitive endoderm genes that were also expressed in Flk1(+) mesoderm cells. (E) Primitive endoderm genes that did not respond to Gata4 in DKO Flk1(+) mesoderm cells. Note that smaller scales for the expression signal are used for Flk1(+) mesoderm cells compared to those for ES cells to present temporal expression changes in (A to E). (F–H) Group 2 genes did not respond to Gata4 in ES cells. (F) Cardiac genes. (G) Genes expressed in mesoderm-derived tissues. (H) Genes expressed in endoderm-derived tissues. “WT+Gata4GR Dex+” and “WT+Gata4GR Dex−”, WT cells expressing Gata4GR with and without Dex, respectively; “DKO+Gata4GR Dex+” and “DKO+Gata4GR Dex−”, DKO cells expressing Gata4GR with and without Dex, respectively. (TIF) [file pgen.1003574.s007.tif]

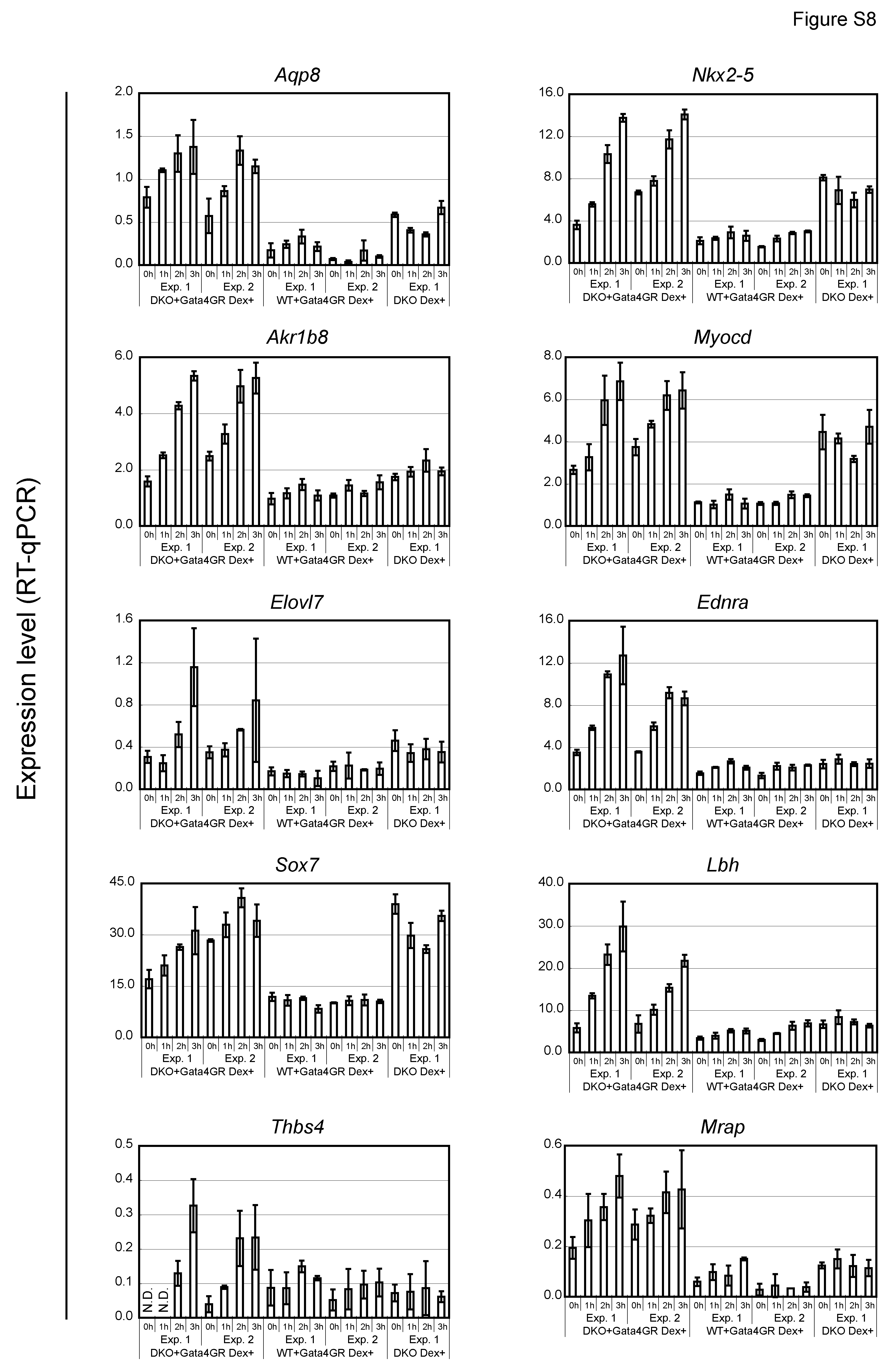

Supplement: Figure S8 — RT-qPCR analysis of Gata4 response genes in Flk1(+) mesoderm cells. ES cells differentiated on OP9 stromal cells were treated with Dex for 0, 1, 2 or 3 hr. The Flk1(+) mesoderm cells were then sorted by flow cytometry, and their gene expression was analyzed using RT-qPCR. The mean values of triplicates for gene expression levels with their standard deviations are shown. “Exp. 1” and “Exp. 2” represent results of independent experiments performed separately. “WT+Gata4GR Dex+”, WT cells expressing Gata4GR with Dex; “DKO+Gata4GR Dex+”, DKO cells expressing Gata4GR with Dex; “DKO Dex+”, DKO cells without the Gata4GR transgene with Dex. (TIF) [file pgen.1003574.s008.tif]

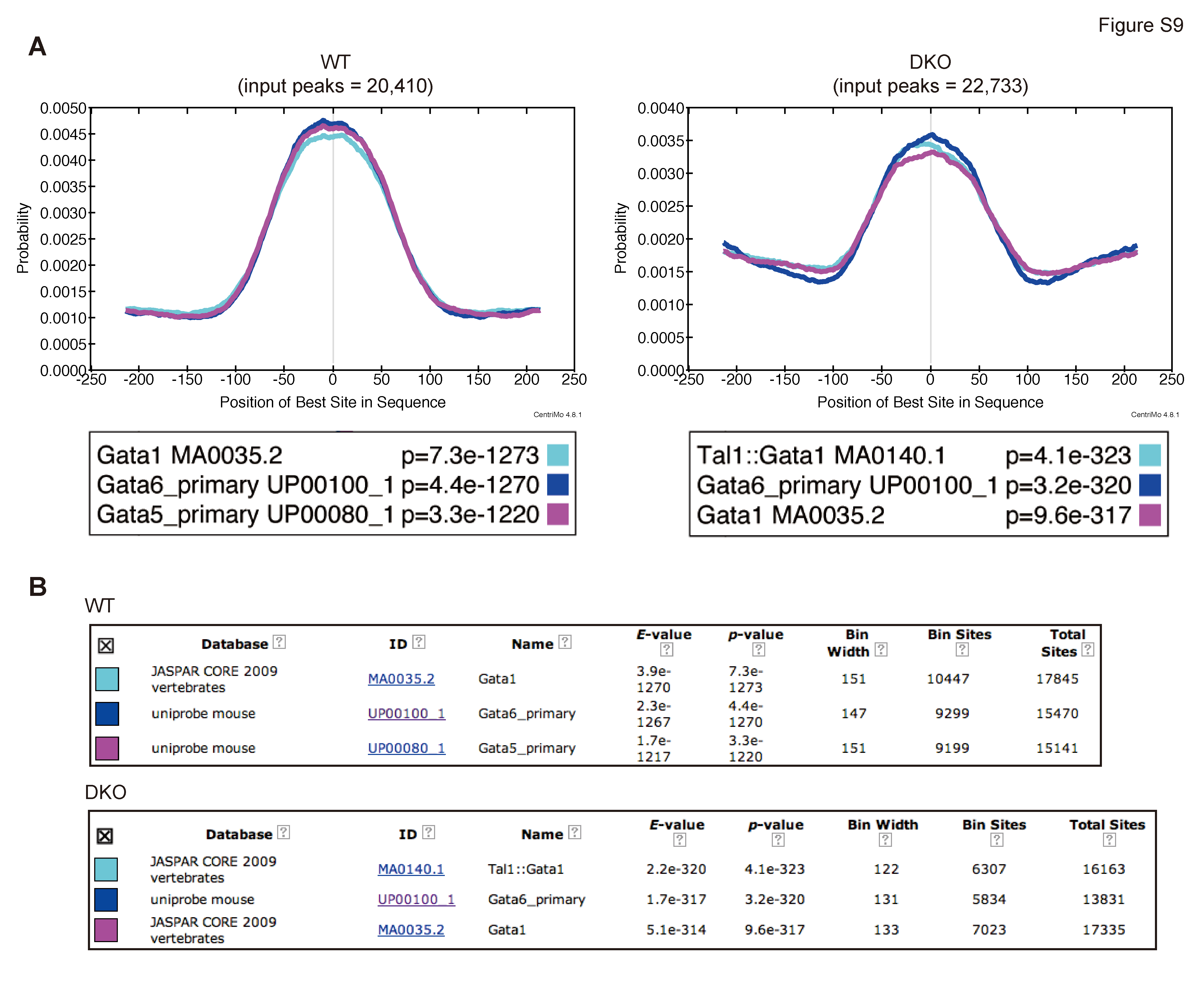

Supplement: Figure S9 — Central motif-enrichment analysis for Gata4-ChIP-seq peaks of WT or DKO Flk1(+) cells with Gata4 activation. ChIP-seq peak regions centered within a 500-bp region for WT (20,410 peaks) or DKO (22,733 peaks) were used as the input for CentriMo. Transcription-factor-binding motifs consisting of vertebrate motifs in the JASPAR CORE database and motifs for mouse transcription factors in the UniPROBE database were used for the motif enrichment analysis. The site-probability curves (A) and the statistics and values (B) for the three most highly ‘centrally enriched’ motifs for WT or DKO are shown. The shape of the site-probability curves and the number of sequences for which the best match to the motif fell in the central region (Bin sites) compared to the number of sequences containing a match to the motif (Total sites) indicated that the Gata motifs were highly centrally enriched in both the WT and DKO Gata4-ChIP-seq peaks. (TIF) [file pgen.1003574.s009.tif]

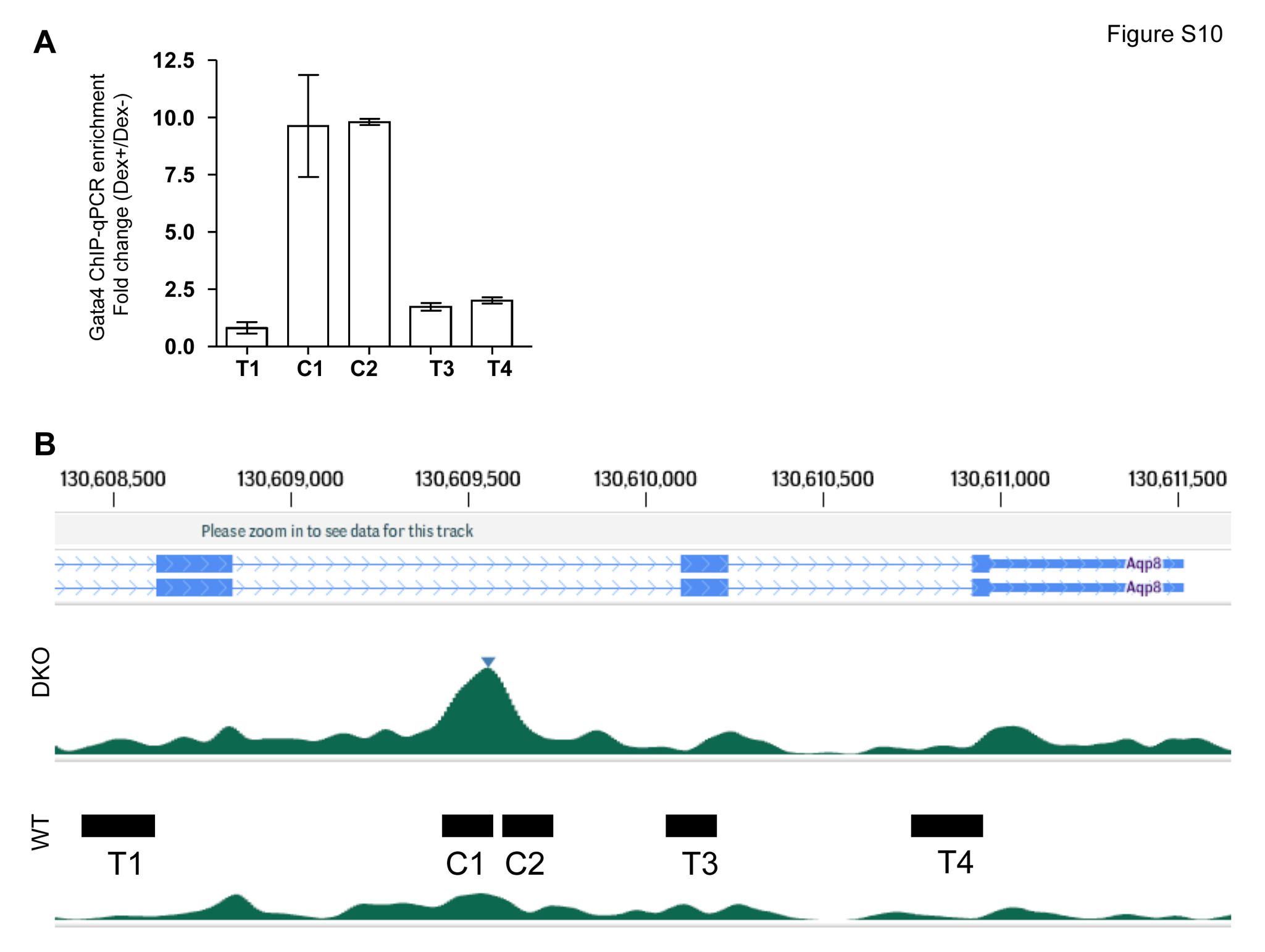

Supplement: Figure S10 — Gata4 ChIP-qPCR analysis at Aqp8 locus for WT ES cells with Gata4 activation. (A) Relative abundance of regions of interest in Gata4 ChIP DNA to input DNA was quantitated by qPCR. Fold enrichment of Gata4 was calculated as the ratio of relative abundance of ChIP DNA in the presence of Dex versus that in the absence of Dex (Dex+/−). (B) The mapped read enrichment from Gata4 ChIP-seq experiments in WT and DKO Flk1(+) cells at Aqp8 locus, an enlarged part of Figure 5B, is shown. The primer-set positions for ChIP-qPCR (T1, C1, C2, T3 and T4) are shown as horizontal bars. (TIF) [file pgen.1003574.s010.tif]

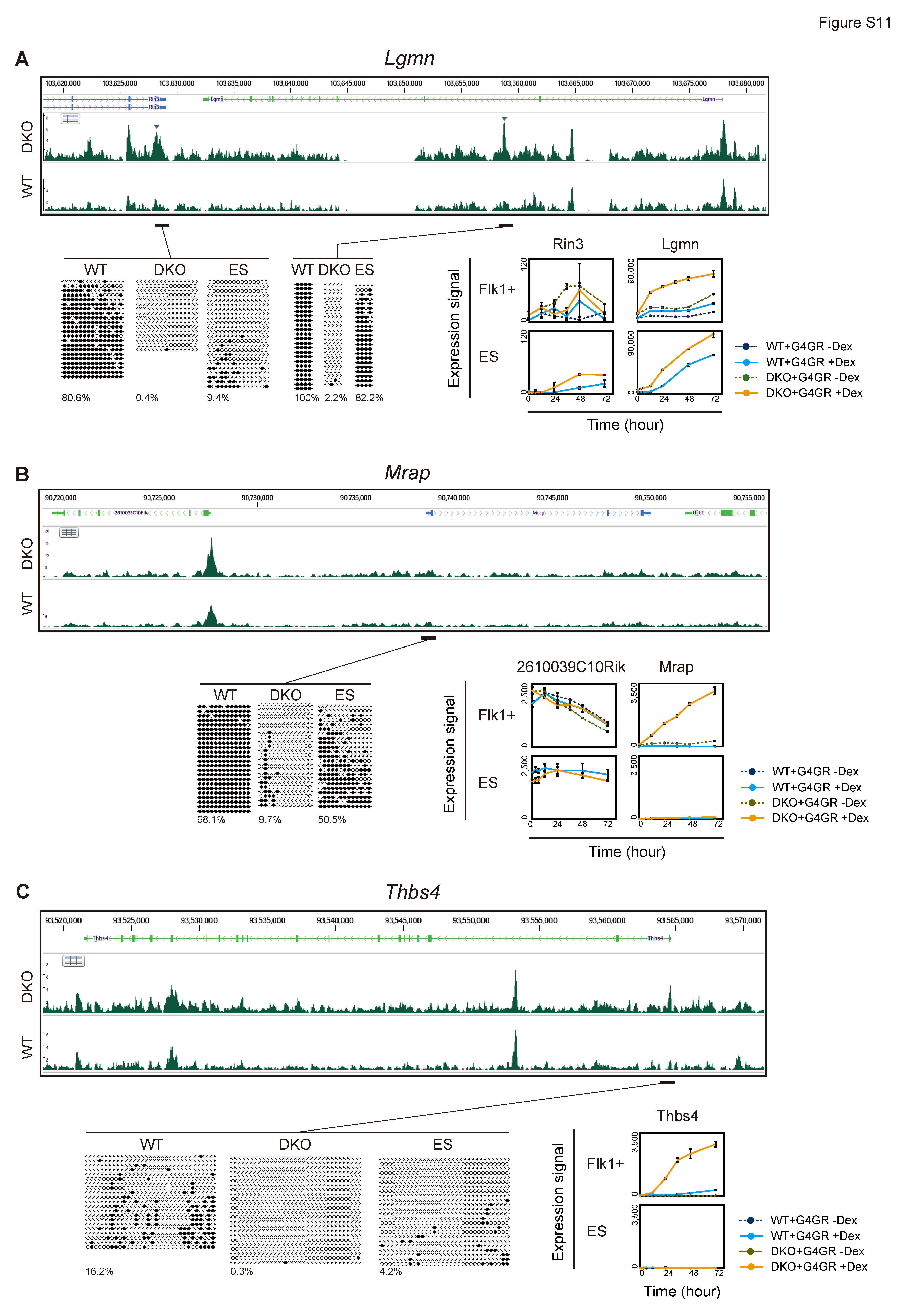

Supplement: Figure S11 — Gata4-binding-site profiles, DNA-methylation states, and Gata4-induced temporal expression changes of Gata4-response genes and neighboring genes. Gata4 ChIP-seq enrichment at the (A) Lgmn, (B) Mrap, and (C) Thbs4 loci in WT or DKO Flk1(+) cells in which Gata4GR was activated by Dex addition is shown. Tracks represent the mapped read enrichment as determined by DNAnexus software. Blue arrowheads mark Gata4 peaks enriched in DKO Flk1(+) cells compared to WT Flk1(+) cells. DNA methylation states at transcription start sites and Gata4-binding sites were analyzed by bisulfite sequencing. Horizontal bars represent the genomic regions subjected to DNA methylation analysis. Line graphs show the temporal expression changes for the indicated genes from microarray data at several time points within 72 hr in WT or DKO Flk1(+) mesoderm cells and ES cells in the presence or absence of Dex. (A) Lgmn and its neighboring gene Rin3 were associated with Gata4 peaks enriched in DKO Flk1(+) cells compared to WT Flk1(+) cells. Rin3 itself did not transcriptionally respond to Gata4, suggesting that the Gata4 peak located in the 3′ region of Rin3 contributes to the Lgmn transcription. Both Gata4 peak regions were methylated in a Dnmt3-dependent manner, and the peak region at Rin3 was de novo methylated during mesoderm commitment. (B) The high-CpG promoter of Mrap was heavily methylated in a Dnmt3-dependent manner. Although Mrap immediately responded to Gata4 in DKO mesoderm cells, no appreciable Gata4 peaks were associated with its proximal genomic region. One Gata4 peak was observed in the neighboring gene, 2610039C10Rik, in both WT and DKO mesoderm cells, while 2610039C10Rik itself did not respond to Gata4. (C) Thbs4 was associated with Gata4 binding at the intronic region in both WT and DKO mesoderm cells, and its promoter region was de novo methylated during mesoderm differentiation. (TIF) [file pgen.1003574.s011.tif]

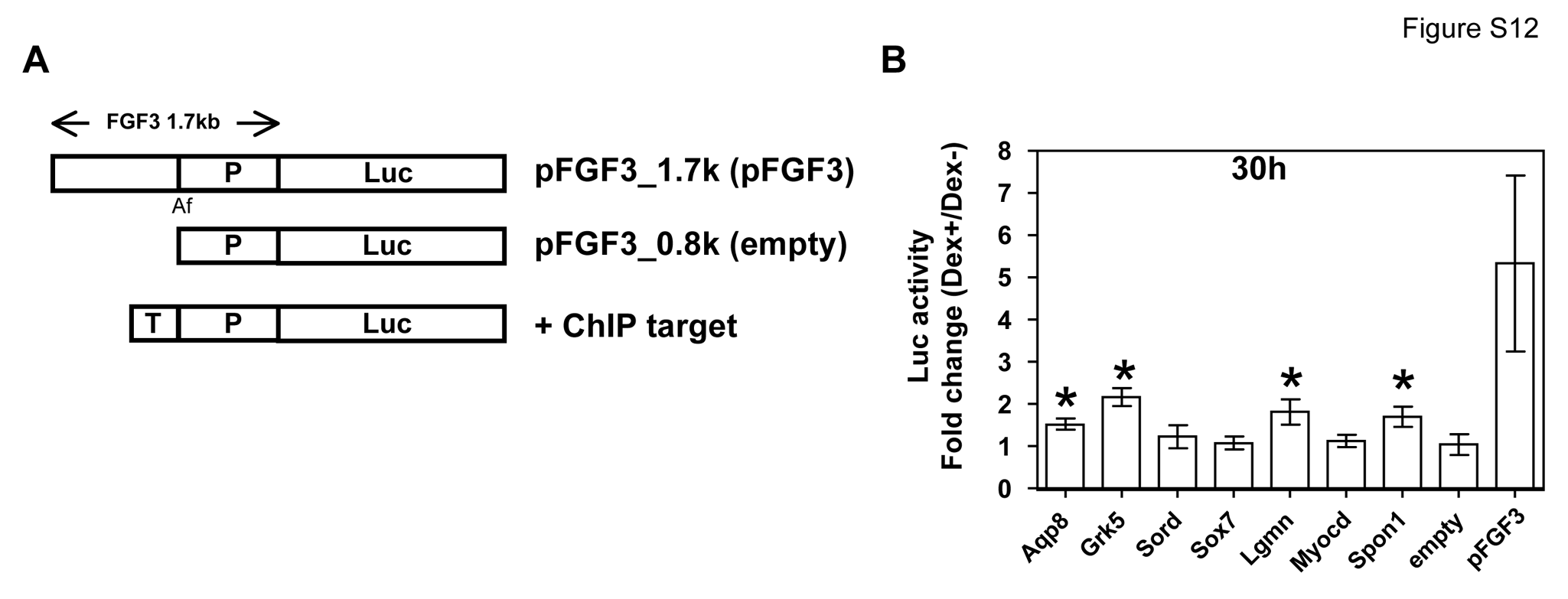

Supplement: Figure S12 — Gata4-dependent enhancer activity of DNA fragments associated with Gata4 ChIP-seq peaks. (A) Schematic diagrams of Gata4-dependent reporter constructs used for luciferase reporter assay. pFGF3_1.7k, Luciferase reporter plasmid containing a 1.7 kb Fgf3 fragment including both Gata4-binding sites and promoter (P). pFGF3_0.8k, Luciferase reporter plasmid containing the 0.8 kb Fgf3 promoter only. ChIP target fragments (0.2–0.3 kb) associated with Gata4 ChIP-seq peaks (T) were inserted to 5′ of the pFGF3_0.8k promoter at the AflII site (Af). (B) Luciferase activity of reporter plasmids containing ChIP-seq peak-associated fragment in response to Gata4 activation by addition of Dex for 30 hr in WT ES cells. Fold changes in luciferase activities in the presence versus the absence of Dex (Dex+/−) were calculated for each reporter construct. The means of triplicates with the standard deviations are shown. pFGF3_1.7k (pFGF3) and pFGF3_0.8k (empty) were used as positive and negative controls, respectively. Asterisks represent statistically significant differences (P<0.05, Student's t-test) compared to the negative control (empty). (TIF) [file pgen.1003574.s012.tif]
