# Supplementary material for: DNA Methylation Restricts Lineage-specific Functions of Transcription Factor Gata4 during Embryonic Stem Cell Differentiation
Source: PLoS Genet. 2013 Jun 27;9(6):e1003574. doi: 10.1371/journal.pgen.1003574 (PMC3694845; doi:10.1371/journal.pgen.1003574)
Supplement: Table S1 — The 20 most significantly enriched gene ontology terms among the differentially expressed genes in WT or Dnmt3a/Dnmt3b-deficient ES or Flk1(+) mesoderm cells with or without Gata4 activation, as categorized in Figure 2B. (PDF) [file pgen.1003574.s013.pdf]

Table S1.
DAVID GO analysis, Biological process 4

1. WT(Dex−) vs DKO (Dex−)

	ES				Flk1+ Dex− 72h			
	up 520 genes (500 DAIVID IDs)				up 941 genes (910 DAVID IDs)			
	Term	Count	Fold Enrichment	PValue	Term	Count	Fold Enrichment	PValue
up	GO:0048513~organ development	79	2.040	4.61E−10	GO:0002684~positive regulation of immune system process	30	3.610	3.80E−09
	GO:0048568~embryonic organ development	22	3.919	2.02E−07	GO:0050778~positive regulation of immune response	24	4.374	4.62E−09
	GO:0007517~muscle organ development	18	4.391	7.16E−07	GO:0050776~regulation of immune response	28	3.634	1.21E−08
	GO:0009888~tissue development	35	2.355	5.29E−06	GO:0001817~regulation of cytokine production	23	4.102	3.50E−08
	GO:0048468~cell development	33	2.319	1.42E−05	GO:0006954~inflammatory response	29	3.195	1.07E−07
	GO:0022008~neurogenesis	30	2.399	2.11E−05	GO:0048584~positive regulation of response to stimulus	26	3.465	1.15E−07
	GO:0048754~branching morphogenesis of a tube	11	5.078	5.77E−05	GO:0070192~chromosome organization involved in meiosis	8	11.665	2.26E−06
	GO:0060537~muscle tissue development	13	4.104	7.68E−05	GO:0007129~synapsis	8	11.665	2.26E−06
	GO:0007399~nervous system development	39	1.961	8.27E−05	GO:0051240~positive regulation of multicellular organismal process	21	3.194	8.78E−06
	GO:0045595~regulation of cell differentiation	23	2.558	9.93E−05	GO:0002697~regulation of immune effector process	15	4.225	1.03E−05
	GO:0060541~respiratory system development	12	4.155	1.45E−04	GO:0048513~organ development	102	1.520	1.12E−05
	GO:0001763~morphogenesis of a branching structure	12	4.122	1.56E−04	GO:0002699~positive regulation of immune effector process	11	5.928	1.13E−05
	GO:0030324~lung development	11	4.255	2.55E−04	GO:0002443~leukocyte mediated immunity	15	4.178	1.17E−05
	GO:0030323~respiratory tube development	11	4.179	2.95E−04	GO:0051094~positive regulation of developmental process	24	2.780	1.75E−05
	GO:0009887~organ morphogenesis	29	2.096	2.99E−04	GO:0002703~regulation of leukocyte mediated immunity	13	4.604	2.02E−05
	GO:0042692~muscle cell differentiation	11	4.037	3.91E−04	GO:0009888~tissue development	49	1.904	2.14E−05
	GO:0045765~regulation of angiogenesis	7	6.679	5.66E−04	GO:0002705~positive regulation of leukocyte mediated immunity	10	6.197	2.33E−05
	GO:0035239~tube morphogenesis	13	3.264	6.48E−04	GO:0002460~adaptive immune response based on somatic recombination of immune receptors built from immunoglobulin superfamily domains	14	4.131	2.87E−05
	GO:0048598~embryonic morphogenesis	20	2.392	7.35E−04	GO:0001819~positive regulation of cytokine production	12	4.798	3.20E−05
	GO:0051093~negative regulation of developmental process	15	2.850	8.22E−04	GO:0002474~antigen processing and presentation of peptide antigen via MHC class I	7	10.207	3.51E−05
down	down 32 genes (30 genes w/o Dnmt3a/b) (29 DAVID IDs)				down 380 genes (378 genes w/o Dnmt3a/b) (369 DAVID IDs)			
	Term	Count	Fold Enrichment	PValue	Term	Count	Fold Enrichment	PValue
	GO:0042127~regulation of cell proliferation	4	8.880	6.61E−03	GO:0048513~organ development	60	2.107	1.94E−08
	GO:0048870~cell motility	3	12.617	1.87E−02	GO:0051252~regulation of RNA metabolic process	53	2.080	2.85E−07
	GO:0000902~cell morphogenesis	3	11.596	2.19E−02	GO:0007399~nervous system development	35	2.393	3.04E−06
	GO:0032989~cellular component morphogenesis	3	10.208	2.78E−02	GO:0015671~oxygen transport	5	26.541	2.47E−05
	GO:0045595~regulation of cell differentiation	3	9.283	3.31E−02	GO:0009887~organ morphogenesis	26	2.556	2.82E−05
	GO:0048522~positive regulation of cellular process	4	3.619	7.09E−02	GO:0009888~tissue development	27	2.471	3.35E−05
					GO:0048598~embryonic morphogenesis	19	3.090	4.40E−05
					GO:0015669~gas transport	5	22.458	5.21E−05
					GO:0010628~positive regulation of gene expression	22	2.632	9.30E−05
					GO:0010557~positive regulation of macromolecule biosynthetic process	23	2.534	1.07E−04
					GO:0048468~cell development	25	2.389	1.21E−04
					GO:0035107~appendage morphogenesis	10	5.077	1.52E−04
					GO:0035108~limb morphogenesis	10	5.077	1.52E−04
					GO:0045935~positive regulation of nucleobase, nucleoside, nucleotide and nucleic acid metabolic process	22	2.519	1.72E−04
					GO:0031328~positive regulation of cellular biosynthetic process	23	2.433	1.91E−04
					GO:0060173~limb development	10	4.907	1.98E−04
					GO:0009891~positive regulation of biosynthetic process	23	2.411	2.17E−04
					GO:0048522~positive regulation of cellular process	41	1.814	2.20E−04
					GO:0035113~embryonic appendage morphogenesis	9	5.418	2.48E−04
					GO:0051173~positive regulation of nitrogen compound metabolic process	22	2.442	2.61E−04

2. WT(Dex−) vs WT (Dex+)

	ES + Dex 0/72h				Flk1+ Dex−/+ 72h			
	up 1096 genes (1087 DAIVID IDs)				up 158 genes (155 DAVID IDs)			
	Term	Count	Fold Enrichment	PValue	Term	Count	Fold Enrichment	PValue
up	GO:0048513~organ development	163	1.928	1.27E−17	GO:0007507~heart development	14	7.364	4.74E−08
	GO:0048568~embryonic organ development	43	3.509	1.57E−12	GO:0003007~heart morphogenesis	8	12.681	2.87E−06
	GO:0001944~vasculature development	39	3.068	1.14E−09	GO:0048513~organ development	33	2.328	4.36E−06
	GO:0048598~embryonic morphogenesis	47	2.575	5.99E−09	GO:0009888~tissue development	18	3.310	2.39E−05
	GO:0001501~skeletal system development	40	2.760	1.44E−08	GO:0048522~positive regulation of cellular process	26	2.311	8.45E−05
	GO:0048646~anatomical structure formation involved in morphogenesis	46	2.479	2.91E−08	GO:0060541~respiratory system development	8	7.568	8.49E−05
	GO:0048523~negative regulation of cellular process	104	1.716	3.80E−08	GO:0009887~organ morphogenesis	16	3.160	1.37E−04
	GO:0009888~tissue development	66	2.035	5.14E−08	GO:0048729~tissue morphogenesis	10	4.929	1.83E−04
	GO:0048562~embryonic organ morphogenesis	27	3.298	1.45E−07	GO:0009952~anterior/posterior pattern formation	8	6.134	3.13E−04
	GO:0043009~chordate embryonic development	48	2.242	2.94E−07	GO:0030324~lung development	7	7.398	3.50E−04
	GO:0009792~embryonic development ending in birth or egg hatching	48	2.221	3.88E−07	GO:0042573~retinoic acid metabolic process	4	27.601	3.66E−04
	GO:0048514~blood vessel morphogenesis	29	2.881	8.17E−07	GO:0030323~respiratory tube development	7	7.267	3.85E−04
	GO:0000904~cell morphogenesis involved in differentiation	30	2.783	1.05E−06	GO:0042692~muscle cell differentiation	7	7.018	4.64E−04
	GO:0009887~organ morphogenesis	59	1.954	1.14E−06	GO:0009799~determination of symmetry	5	13.330	5.05E−04
	GO:0001569~patterning of blood vessels	10	8.195	1.40E−06	GO:0009855~determination of bilateral symmetry	5	13.330	5.05E−04
	GO:0048522~positive regulation of cellular process	106	1.579	1.64E−06	GO:0060537~muscle tissue development	7	6.038	1.03E−03
	GO:0001525~angiogenesis	22	3.253	3.31E−06	GO:0001944~vasculature development	9	4.223	1.26E−03
	GO:0042127~regulation of cell proliferation	53	1.938	5.64E−06	GO:0016101~diterpenoid metabolic process	4	16.758	1.65E−03
	GO:0007399~nervous system development	74	1.704	6.75E−06	GO:0060429~epithelium development	9	3.896	2.09E−03
	GO:0007417~central nervous system development	40	2.132	1.14E−05	GO:0003002~regionalization	8	4.385	2.24E−03
down	down 1014 genes (986 DAVID IDs)				down 50 genes (48 DAVID IDs)			
	Term	Count	Fold Enrichment	PValue	Term	Count	Fold Enrichment	PValue
	GO:0051093~negative regulation of developmental process	31	3.086	7.43E−08	GO:0007399~nervous system development	10	4.525	1.85E−04
	GO:0048513~organ development	117	1.583	2.89E−07	GO:0007417~central nervous system development	7	7.330	2.75E−04
	GO:0045596~negative regulation of cell differentiation	26	3.214	4.73E−07	GO:0022008~neurogenesis	8	5.757	3.10E−04
	GO:0045595~regulation of cell differentiation	41	2.390	5.17E−07	GO:0030182~neuron differentiation	7	6.779	4.17E−04
	GO:0042127~regulation of cell proliferation	51	2.133	5.59E−07	GO:0007420~brain development	6	8.050	7.01E−04
	GO:0048522~positive regulation of cellular process	95	1.619	2.07E−06	GO:0000904~cell morphogenesis involved in differentiation	5	9.113	1.87E−03
	GO:0048468~cell development	53	1.951	4.47E−06	GO:0048468~cell development	7	4.427	3.73E−03
	GO:0051252~regulation of RNA metabolic process	99	1.497	3.26E−05	GO:0007423~sensory organ development	5	7.518	3.75E−03
	GO:0048864~stem cell development	8	7.825	4.30E−05	GO:0050767~regulation of neurogenesis	4	11.709	4.34E−03
	GO:0048523~negative regulation of cellular process	82	1.548	6.15E−05	GO:0048666~neuron development	5	6.617	5.89E−03
	GO:0010556~regulation of macromolecule biosynthetic process	143	1.351	6.89E−05	GO:0051960~regulation of nervous system development	4	10.444	5.97E−03
	GO:0060255~regulation of macromolecule metabolic process	158	1.322	7.40E−05	GO:0048513~organ development	11	2.556	6.05E−03
	GO:0031326~regulation of cellular biosynthetic process	147	1.340	7.75E−05	GO:0010720~positive regulation of cell development	3	24.665	6.16E−03
	GO:0048646~anatomical structure formation involved in morphogenesis	34	2.096	8.43E−05	GO:0000902~cell morphogenesis	5	6.253	7.17E−03
	GO:0009889~regulation of biosynthetic process	147	1.336	9.42E−05	GO:0060284~regulation of cell development	4	9.721	7.27E−03
	GO:0007399~nervous system development	62	1.633	1.44E−04	GO:0009968~negative regulation of signal transduction	4	9.039	8.88E−03
	GO:0009891~positive regulation of biosynthetic process	45	1.817	1.47E−04	GO:0045597~positive regulation of cell differentiation	4	8.832	9.45E−03
	GO:0008284~positive regulation of cell proliferation	28	2.218	1.62E−04	GO:0048812~neuron projection morphogenesis	4	8.782	9.60E−03
	GO:0010557~positive regulation of macromolecule biosynthetic process	43	1.825	1.92E−04	GO:0032989~cellular component morphogenesis	5	5.504	1.11E−02
	GO:0045619~regulation of lymphocyte differentiation	11	4.267	2.08E−04	GO:0010648~negative regulation of cell communication	4	8.310	1.12E−02

Table S1 (continued).

3. DKO(Dex-) vs DKO (Dex+)

	ES Dex+ 0/72h				Flk1+ Dex-/+ 72h			
	up 1021 genes (1007 DAIVID IDs)				up 531 genes (528 DAVID IDs)			
	Term	Count	Fold Enrichment	PValue	Term	Count	Fold Enrichment	PValue
up	GO:0048513~organ development	135	1.769	1.51E-11	GO:0048513~organ development	78	1.845	6.95E-08
	GO:0042127~regulation of cell proliferation	53	2.146	2.60E-07	GO:0008016~regulation of heart contraction	9	7.080	3.32E-05
	GO:0043085~positive regulation of catalytic activity	32	2.671	1.10E-06	GO:0006936~muscle contraction	9	5.900	1.27E-04
	GO:0045595~regulation of cell differentiation	41	2.314	1.17E-06	GO:0007507~heart development	16	2.822	5.70E-04
	GO:0001944~vasculature development	31	2.702	1.34E-06	GO:0048469~cell maturation	9	4.720	6.03E-04
	GO:0009888~tissue development	57	1.947	2.00E-06	GO:0044057~regulation of system process	14	2.740	1.86E-03
	GO:0048754~branching morphogenesis of a tube	17	3.983	4.41E-06	GO:0008015~blood circulation	10	3.544	2.03E-03
	GO:0048568~embryonic organ development	29	2.622	5.63E-06	GO:0015671~oxygen transport	4	14.304	2.29E-03
	GO:0051174~regulation of phosphorus metabolic process	33	2.389	8.13E-06	GO:0060537~muscle tissue development	11	3.182	2.42E-03
	GO:0009887~organ morphogenesis	52	1.907	1.09E-05	GO:0003007~heart morphogenesis	8	4.252	2.65E-03
	GO:0007507~heart development	27	2.638	1.14E-05	GO:0015669~gas transport	4	12.103	3.82E-03
	GO:0048523~negative regulation of cellular process	87	1.590	1.28E-05	GO:0060541~respiratory system development	10	3.172	4.29E-03
	GO:0051338~regulation of transferase activity	25	2.737	1.39E-05	GO:0001944~vasculature development	15	2.360	4.70E-03
	GO:0001763~morphogenesis of a branching structure	19	3.312	1.51E-05	GO:0009887~organ morphogenesis	27	1.788	4.79E-03
	GO:0048522~positive regulation of cellular process	93	1.535	2.43E-05	GO:0007517~muscle organ development	12	2.682	5.26E-03
	GO:0048646~anatomical structure formation involved in morphogenesis	36	2.149	2.92E-05	GO:0043436~oxoacid metabolic process	23	1.889	5.34E-03
	GO:0001525~angiogenesis	19	3.113	3.55E-05	GO:0009888~tissue development	28	1.726	6.42E-03
	GO:0048729~tissue morphogenesis	27	2.472	3.58E-05	GO:0048468~cell development	27	1.738	6.86E-03
	GO:0048514~blood vessel morphogenesis	24	2.641	3.83E-05	GO:0030324~lung development	9	3.189	7.22E-03
	GO:0000904~cell morphogenesis involved in differentiation	25	2.569	3.99E-05	GO:0030323~respiratory tube development	9	3.133	8.01E-03
down	down 1353 genes (1308 DAVID IDs)				down 609 genes (601 DAVID IDs)			
	Term	Count	Fold Enrichment	PValue	Term	Count	Fold Enrichment	PValue
	GO:0048513~organ development	172	1.724	1.71E-13	GO:0048513~organ development	110	2.189	6.29E-16
	GO:0007399~nervous system development	103	2.011	7.28E-12	GO:0009888~tissue development	59	3.060	3.01E-14
	GO:0051093~negative regulation of developmental process	43	3.172	3.73E-11	GO:0001501~skeletal system development	31	3.600	2.44E-09
	GO:0022008~neurogenesis	72	2.235	1.80E-10	GO:0009887~organ morphogenesis	46	2.563	1.06E-08
	GO:0045595~regulation of cell differentiation	57	2.462	5.36E-10	GO:0048523~negative regulation of cellular process	69	1.916	1.70E-07
	GO:0009888~tissue development	76	1.986	1.15E-08	GO:0060429~epithelium development	27	3.297	1.89E-07
	GO:0030182~neuron differentiation	55	2.298	1.34E-08	GO:0022603~regulation of anatomical structure morphogenesis	20	3.539	3.72E-06
	GO:0048468~cell development	73	1.992	2.06E-08	GO:0048598~embryonic morphogenesis	29	2.673	4.19E-06
	GO:0048522~positive regulation of cellular process	128	1.617	2.88E-08	GO:0007399~nervous system development	51	1.976	4.47E-06
	GO:0045596~negative regulation of cell differentiation	33	3.023	3.27E-08	GO:0051093~negative regulation of developmental process	22	3.221	4.83E-06
	GO:0042127~regulation of cell proliferation	66	2.045	4.06E-08	GO:0035113~embryonic appendage morphogenesis	14	4.776	6.54E-06
	GO:0048666~neuron development	43	2.455	1.04E-07	GO:0003002~regionalization	21	3.247	7.43E-06
	GO:0048523~negative regulation of cellular process	114	1.595	4.07E-07	GO:0022612~gland morphogenesis	13	5.122	7.58E-06
	GO:0022603~regulation of anatomical structure morphogenesis	31	2.764	6.88E-07	GO:0048705~skeletal system morphogenesis	16	4.073	8.48E-06
	GO:0045165~cell fate commitment	26	2.949	1.95E-06	GO:0030182~neuron differentiation	30	2.488	1.12E-05
	GO:0048646~anatomical structure formation involved in morphogenesis	46	2.101	3.01E-06	GO:0060173~limb development	15	4.171	1.35E-05
	GO:0048864~stem cell development	10	7.249	3.62E-06	GO:0045595~regulation of cell differentiation	29	2.486	1.64E-05
	GO:0009952~anterior/posterior pattern formation	26	2.833	4.12E-06	GO:0042330~taxis	14	4.251	2.36E-05
	GO:0009887~organ morphogenesis	64	1.796	6.18E-06	GO:0006935~chemotaxis	14	4.251	2.36E-05
	GO:0048863~stem cell differentiation	11	5.916	7.87E-06	GO:0001763~morphogenesis of a branching structure	15	3.971	2.38E-05

4. WT(Dex+) vs DKO (Dex+)

	ES Dex+ 72h				Flk1+ Dex+ 72h			
	up 368 genes (357 DAIVID IDs)				up 896 genes (866 DAVID IDs)			
	Term	Count	Fold Enrichment	PValue	Term	Count	Fold Enrichment	PValue
up	GO:0060415~muscle tissue morphogenesis	4	17.275	1.46E-03	GO:0070192~chromosome organization involved in meiosis	8	12.695	1.28E-06
	GO:0022603~regulation of anatomical structure morphogenesis	9	3.741	2.78E-03	GO:0007129~synapsis	8	12.695	1.28E-06
	GO:0002834~regulation of response to tumor cell	3	33.317	3.27E-03	GO:0050776~regulation of immune response	22	3.107	8.06E-06
	GO:0002837~regulation of immune response to tumor cell	3	33.317	3.27E-03	GO:0007127~meiosis I	9	6.937	3.22E-05
	GO:0002833~positive regulation of response to biotic stimulus	3	33.317	3.27E-03	GO:0050778~positive regulation of immune response	17	3.372	4.20E-05
	GO:0002839~positive regulation of immune response to tumor cell	3	33.317	3.27E-03	GO:0002684~positive regulation of immune system process	20	2.619	2.33E-04
	GO:0002836~positive regulation of response to tumor cell	3	33.317	3.27E-03	GO:0006954~inflammatory response	21	2.518	2.63E-04
	GO:0051240~positive regulation of multicellular organismal process	8	3.815	4.96E-03	GO:0002474~antigen processing and presentation of peptide antigen via MHC class I	6	9.521	2.90E-04
	GO:0031349~positive regulation of defense response	5	6.819	6.02E-03	GO:0048477~oogenesis	8	5.534	4.99E-04
	GO:0006954~inflammatory response	9	3.110	8.33E-03	GO:0048584~positive regulation of response to stimulus	18	2.611	5.44E-04
	GO:0045089~positive regulation of innate immune response	4	8.638	1.08E-02	GO:0006958~complement activation, classical pathway	7	6.295	6.92E-04
	GO:0048729~tissue morphogenesis	9	2.940	1.15E-02	GO:0007292~female gamete generation	9	4.336	9.86E-04
	GO:0002831~regulation of response to biotic stimulus	3	17.940	1.16E-02	GO:0002253~activation of immune response	11	3.451	1.25E-03
	GO:0002705~positive regulation of leukocyte mediated immunity	4	7.774	1.44E-02	GO:0016338~calcium-independent cell-cell adhesion	6	7.038	1.31E-03
	GO:0003007~heart morphogenesis	5	5.253	1.48E-02	GO:0002699~positive regulation of immune effector process	8	4.692	1.39E-03
	GO:0045954~positive regulation of natural killer cell mediated cytotoxicity	3	15.548	1.53E-02	GO:0031347~regulation of defense response	12	3.143	1.47E-03
	GO:0045429~positive regulation of nitric oxide biosynthetic process	3	15.548	1.53E-02	GO:0051327~M phase of meiotic cell cycle	11	3.372	1.49E-03
	GO:0051130~positive regulation of cellular component organization	6	3.823	2.02E-02	GO:0007126~meiosis	11	3.372	1.49E-03
	GO:0002699~positive regulation of immune effector process	4	6.760	2.09E-02	GO:0002455~humoral immune response mediated by circulating immunoglobulin	7	5.395	1.62E-03
	GO:0045088~regulation of innate immune response	4	6.760	2.09E-02	GO:0002443~leukocyte mediated immunity	11	3.334	1.62E-03
down	down 277 genes (275 genes w/o Dnmt3a/b) (264 DAVID IDs)				down 539 genes (529 DAVID IDs)			
	Term	Count	Fold Enrichment	PValue	Term	Count	Fold Enrichment	PValue
	GO:0048646~anatomical structure formation involved in morphogenesis	23	5.110	6.39E-10	GO:0048513~organ development	98	2.264	2.82E-15
	GO:0001829~trophectodermal cell differentiation	7	35.481	2.23E-08	GO:0007399~nervous system development	55	2.474	8.11E-10
	GO:0048513~organ development	46	2.243	1.46E-07	GO:0009888~tissue development	43	2.589	2.50E-08
	GO:0001825~blastocyst formation	7	25.804	1.96E-07	GO:0001501~skeletal system development	27	3.639	2.64E-08
	GO:0019827~stem cell maintenance	7	25.804	1.96E-07	GO:0009887~organ morphogenesis	41	2.652	2.94E-08
	GO:0048864~stem cell development	7	24.682	2.62E-07	GO:0051093~negative regulation of developmental process	23	3.910	1.03E-07
	GO:0007498~mesoderm development	9	12.371	5.57E-07	GO:0048522~positive regulation of cellular process	67	1.950	1.17E-07
	GO:0048863~stem cell differentiation	7	18.313	1.76E-06	GO:0048562~embryonic organ morphogenesis	19	4.533	1.93E-07
	GO:0048568~embryonic organ development	14	4.711	8.31E-06	GO:0048598~embryonic morphogenesis	29	3.103	2.15E-07
	GO:0048598~embryonic morphogenesis	17	3.840	8.52E-06	GO:0048705~skeletal system morphogenesis	17	5.024	2.48E-07
	GO:0043009~chordate embryonic development	18	3.467	1.60E-05	GO:0048568~embryonic organ development	23	3.666	3.18E-07
	GO:0009792~embryonic development ending in birth or egg hatching	18	3.435	1.81E-05	GO:0048732~gland development	20	3.900	8.94E-07
	GO:0009952~anterior/posterior pattern formation	11	5.831	1.84E-05	GO:0045596~negative regulation of cell differentiation	19	4.010	1.20E-06
	GO:0051093~negative regulation of developmental process	13	4.665	2.19E-05	GO:0001655~urogenital system development	17	4.473	1.22E-06
	GO:0048468~cell development	21	2.787	5.76E-05	GO:0007411~axon guidance	14	5.488	1.42E-06
	GO:0003002~regionalization	12	4.548	6.52E-05	GO:0022612~gland morphogenesis	13	5.945	1.63E-06
	GO:0009887~organ morphogenesis	20	2.731	1.21E-04	GO:0030182~neuron differentiation	29	2.792	1.81E-06
	GO:0042472~inner ear morphogenesis	7	8.473	1.66E-04	GO:0001763~morphogenesis of a branching structure	15	4.610	4.38E-06
	GO:0001944~vasculature development	12	3.893	2.57E-04	GO:0001944~vasculature development	21	3.227	8.36E-06
	GO:0000904~cell morphogenesis involved in differentiation	11	4.208	2.82E-04	GO:0045595~regulation of cell differentiation	27	2.687	8.85E-06
