# Supplementary material for: DNA Methylation Restricts Lineage-specific Functions of Transcription Factor Gata4 during Embryonic Stem Cell Differentiation
Source: PLoS Genet. 2013 Jun 27;9(6):e1003574. doi: 10.1371/journal.pgen.1003574 (PMC3694845; doi:10.1371/journal.pgen.1003574)
Supplement: Table S2 — The 20 most significantly enriched gene ontology terms among the upregulated genes in Dnmt3a/Dnmt3b-deficient Flk1(+) mesoderm cells with Gata4 activation, as categorized in Figure 2C. (PDF) [file pgen.1003574.s014.pdf]

Table S2.
DAVID GO analysis, Biological Process 4

	[WT (Dex+) < DKO (Dex+)] AND [DKO (Dex−) < DKO (Dex+)] 710 genes (701 DAVID IDs)				[WT (Dex+) < DKO (Dex+)] NOT [DKO (Dex−) < DKO (Dex+)] 974 genes (944 DAVID IDs)			
	Term	Count	Fold Enrichment	PValue	Term	Count	Fold Enrichment	PValue
up	GO:0007507~heart development	24	3.236	1.44E-06	GO:0050778~positive regulation of immune response	19	3.463	8.56E-06
	GO:0008016~regulation of heart contraction	11	6.614	4.57E-06	GO:0002684~positive regulation of immune system process	24	2.888	9.37E-06
	GO:0048513~organ development	86	1.555	2.54E-05	GO:0007129~synapsis	7	10.207	3.51E-05
	GO:0042692~muscle cell differentiation	14	3.597	1.33E-04	GO:0070192~chromosome organization involved in meiosis	7	10.207	3.51E-05
	GO:0060541~respiratory system development	13	3.152	8.49E-04	GO:0048584~positive regulation of response to stimulus	21	2.799	6.09E-05
	GO:0044106~cellular amine metabolic process	20	2.349	9.12E-04	GO:0050776~regulation of immune response	21	2.725	8.83E-05
	GO:0030324~lung development	12	3.250	1.14E-03	GO:0045087~innate immune response	15	3.475	9.63E-05
	GO:0048523~negative regulation of cellular process	60	1.513	1.15E-03	GO:0031343~positive regulation of cell killing	7	7.544	2.33E-04
	GO:0030323~respiratory tube development	12	3.193	1.32E-03	GO:0001912~positive regulation of leukocyte mediated cytotoxicity	7	7.544	2.33E-04
	GO:0043436~oxoacid metabolic process	30	1.883	1.37E-03	GO:0001916~positive regulation of T cell mediated cytotoxicity	5	13.771	2.79E-04
	GO:0060537~muscle tissue development	13	2.874	1.89E-03	GO:0006749~glutathione metabolic process	7	7.230	3.00E-04
	GO:0007517~muscle organ development	15	2.562	2.21E-03	GO:0007127~meiosis I	8	5.666	4.15E-04
	GO:0050727~regulation of inflammatory response	8	4.220	2.67E-03	GO:0002455~humoral immune response mediated by circulating immunoglobulin	8	5.666	4.15E-04
	GO:0006873~cellular ion homeostasis	19	2.189	2.78E-03	GO:0002474~antigen processing and presentation of peptide antigen via MHC class I	6	8.749	4.30E-04
	GO:0044057~regulation of system process	16	2.393	2.91E-03	GO:0002460~adaptive immune response based on somatic recombination of immune receptors built from immunoglobulin superfamily domains	12	3.541	5.26E-04
	GO:0003007~heart morphogenesis	9	3.656	3.06E-03	GO:0001914~regulation of T cell mediated cytotoxicity	5	11.268	6.84E-04
	GO:0048469~cell maturation	9	3.608	3.33E-03	GO:0002449~lymphocyte mediated immunity	11	3.588	9.00E-04
	GO:0030334~regulation of cell migration	10	3.268	3.42E-03	GO:0006958~complement activation, classical pathway	7	5.784	1.08E-03
	GO:0006936~muscle contraction	8	4.009	3.58E-03	GO:0001910~regulation of leukocyte mediated cytotoxicity	7	5.784	1.08E-03
	GO:0008015~blood circulation	11	2.979	3.80E-03	GO:0030855~epithelial cell differentiation	14	2.821	1.35E-03
