# Supplementary material for: DNA Methylation Restricts Lineage-specific Functions of Transcription Factor Gata4 during Embryonic Stem Cell Differentiation
Source: PLoS Genet. 2013 Jun 27;9(6):e1003574. doi: 10.1371/journal.pgen.1003574 (PMC3694845; doi:10.1371/journal.pgen.1003574)
Supplement: Table S4 — Primers and PCR conditions used in this study. (PDF) [file pgen.1003574.s016.pdf]

Table S4. Primers and PCR conditions used in this study

	Gene	forward	reverse	PCR conditions*
RT-PCR	*Brachyury (T)*	ATGCCAAAGAAAGAAACGAC	AGAGGCTGTAGAACATGATT	A
	Bmp4	TGTGAGGAGTTTCCATCACG	TTATTCTTCTTCCTGGACCG	A
	Isl1	AGCAAGAACGACTTCGTGATG	GACTGAGAGGGTCTCCAGCTC	A
	Gata4	GCCTGTATGTAATGCCTGCG	CCGAGCAGGAATTTGAAGAGG	A
	Gata6	GCAATGCATGCGGTCTCTAC	CTCTTGGTAGCACCAGCTCA	A
	Foxa2	CCCTACGCCAACATGAACTCG	GTTCTGCCGGTAGAAAGGGA	A
	Dab2	GGCAACAGGCTGAACCATTAGT	TTGGTGTCGATTTCAGAGTTTAGAT	A
	Gapdh	ATGGTGAAGGTCGGTGTGAACGGATTTGGC	GCATCGAAGGTGGAAGAGTGGGAGTTGCTG	A
	SMA	ACGGCCGCCTCCTCTTCCTC	GCCCAGCTTCGTCGTATTCC	A
	Sox7	ATGCTGGGAAAGTCATGGAAG	CGTGTTCTGGTCACGAGAGA	A
	Fgf3	ACAGCGCCTATAGCATCCTG	CGCACCATCTCATGGTCCTT	A
	Gata4-GR	CCTGTCACTCAGACATCGCA	GCGTAGTCATGATCCTCCAAGT	A
	Afp	TCGTATTCCAACAGGAGG	AGGCTTTTGCTTCACCAG	A
	Hnf4	TGCCCTCTCACCTCAGCAATG	CCCCTCAGCACACGGTTTTG	A
RT-qPCR	*Aqp8*	GAAGAACTGCTGGCGACGTT	GGTCAGCTGCTGTCATGGTG	B
	Akr1b8	TGATGCACACTTCCCTCCTG	TTGCTCGCAACAGTCTGTCC	B
	Elovl7	TGTGTGTCCAGCCTGGGTTA	CAATTCCGTTGGGTCTTTGC	B
	Sox7	CCCCCTTACTCACCGGAGTT	ATGGGGGACATCCAGAAACA	B
	Thbs4	CCAGGAGTTCCAGACCCAGA	TTGGTGTTTGAGAAGGCAGTTG	B
	Nkx2-5	TGGGAAAGCTCCCACTATGC	GGGATGGATCGGAGAAAGGT	B
	Myocd	CGGAATCTCCTTGGGAAACA	GGCCCACTGGAGGTAAGGTT	B
	Ednra	GCGCATGTCATTTCCAACAC	AGAACTGCACGGAAGCTGGT	B
	Lbh	CAGCCACCAGCCTTTGTTTT	TGAAGCATCTGGGGAATGGT	B
	Mrap	GACCAGTGGTTAACGCAGCA	GCTCTGTCTCCAGGCTCACC	B
	Gapdh	AACTTTGGCATTGTGGAAGG	GGATGCAGGGATGATGTTCT	B
	Rps21	GACCACAGGCCGGTTTAATG	CCCCGCAGATGCCATAGG	B
	Rps27a	GAGACTTCGGGGTGGTGCTA	TTTCAGCACAGCCAACTTAACC	B
Bisulfite sequencing	*Mrap*	GGTAAAAGTTGGGGATATATTTAGTTAAGA	TAAACAAACCTCCAAAAACTACTAAAAAC	C
	Aqp8_pro1	ATATTTTTGGTGAATGTTTTTAGTTTTTTA	ACCCACAAATTCCACTATACATAATTATAC	D
	Aqp8_233	TTATTTTAGTGGATATGTAGAGAGGTTAGA	CCAAAAACTATAAAAAAACCTTACTTAACT	C
	Sox7_250	GTTTTTGAGGGTAAAATTAGGTTTTAAA	CTCTCTAATCTCAAAATCCCTATAAAATAACT	C
	Sox7_222	GTTATAAATGTTTGTGGGTTTAATTTTAG	CCTATATATCCCAACTACTTCCTTACAATA	C
	Sox7_pro1	GTTTTATTGTAAGGAAGTAGTTGGGATATA	ACTATTATAAACAAATCACACCCATAACTT	D
	Sox7_pro4	GAGGTTGTTGGTTAAGTTGGA	CCACACCATAAAAACATTCATAAAC	D
	Lgmn1_248	TATATTTGTTATTTAAAATATTGAGGAGATGT	TAAAACTAATAAATTTAACAAACCTCAAACC	C
	Lgmn1_1	TAGGATTTTATTTGTGTGTTTTATTTGAA	CTACCACCACAACTTATAAAAAATTAAATT	D
	Thbs4	GTATGGTGATTTTTTTGTTTTTTGAG	CTATAACCCTCTCTACCCAACCTCTAT	D
ChIP-qPCR	*Aqp8_T1*	TAGGGCCCAACACCCATTATC	CGAAGAAGCTGCAAGACTTCC	E
	Aqp8_C1	ACCATCAGGACATGCACATTG	ATATCCACTGAGGTGGCAGTATC	E
	Aqp8_C2	GCTACAGCCTATGGAGTGGATAC	GGGCAGTTCTACTCTGTGAC	E
	Aqp8_T3	GGACCTTCTGAGATGCTGGTAAC	CATGCAGGCTCCAGAGATG	E
	Aqp8_T4	AGAGGACTCAGCTCAGGAAAC	GAAAGACCCAGCTCTCCATTAC	E
Luc assay construct	*Aqp8*	ATCTCGAGTGAAGGCACTGGGTTTGGAGAC	AACTTAAGCCGGGCAGTTCTACTCTGTGAC	F
	Grk5	ATCTCGAGCACTGGGACTAAAGGTATGCATCTCCATT	AACTTAAGGGTTGTAGGTCAAGTTATCTGTGCCTCTC	F
	Sord	ATCTCGAGCACCTCGAAGCAAGAGAGCAACAT	AACTTAAGCTGGGTAAACCCAGGCAGTCAG	F
	Sox7	ATCTCGAGTTCTCCGGACATTTACAGCGTCTCAG	AACTTAAGGCGTGACAGCAAGTCCGGTATC	F
	Lgmn	ATCTCGAGTGGCTGATGAGCCAGCATACTTG	AACTTAAGGTGACCAGCTGTTGGGAGGTTG	F
	Myocd	AACTTAAGAGGCCACCCTCTTTGCTGGTTC	ATCTCGAGTTGTCATCACACTGCCTGACGTGTCTG	F
	Spon1	ATCTCGAGCCATACGGCAGGAGAGGACCAAT	AACTTAAGGGCTATCACTGCAGCGTCAAGTT	F

*PCR conditions

	RT-PCR	
A	1 cycle	95˚C 2min
	21-35 cycle	95˚C 30sec / 58˚C 1min / 72˚C 2min
	1 cycle	72˚C 5min
	RT-qPCR	
B	1 cycle	95˚C 10min
	40 cycle	95˚C 15sec / 60˚C 1min
	Bisulfite-PCR	
C	1 cycle	95˚C 15min
	2 cycle	94˚C 2min / 55˚C 1min / 72˚C 1min
	38 cycle	94˚C 1min / 50˚C 1min / 72˚C 1min
	1 cycle	72˚C 10min
D	1 cycle	95˚C 15min
	2 cycle	94˚C 2min / 55˚C 1min / 72˚C 1min
	38 cycle	94˚C 1min / 55˚C 1min / 72˚C 1min
	1 cycle	72˚C 10min
	ChIP-qPCR	
E	1 cycle	95˚C 2min
	40 cycle	95˚C 15sec / 58˚C 20sec / 72˚C 25sec
	Luc assay construct	
F	1 cycle	95˚C 15min
	40 cycle	94˚C 30sec / 56˚C 30sec / 72˚C 1min
